# The impact of health insurance on maternal and reproductive health service utilization and financial protection in low- and lower middle-income countries: a systematic review of the evidence

**DOI:** 10.1186/s12913-024-10815-5

**Published:** 2024-04-05

**Authors:** Joseph Kazibwe, Phuong Bich Tran, Andrea Hannah Kaiser, Simon Peter Kasagga, Felix Masiye, Björn Ekman, Jesper Sundewall

**Affiliations:** 1 Department of Clinical Sciences, Lund University, Jan Waldenströms Gata, 35205 02 Malmö, Sweden; 2 Department of Family Medicine and Population Health, University of Antwerp, Antwerp, Belgium; 3 Department of Social Science, Makerere University, Kampala, Uganda; 4 Department of Economics, University of Zambia, Lusaka, Zambia; 5 HEARD, University of KwaZulu-Natal, Durban, South Africa

**Keywords:** Health insurance, Impact, Low and lower middle-income countries, Maternal and reproductive health, Financial protection, UHC

## Abstract

**Background:**

Low- and middle-income countries have committed to achieving universal health coverage (UHC) as a means to enhance access to services and improve financial protection. One of the key health financing reforms to achieve UHC is the introduction or expansion of health insurance to enhance access to basic health services, including maternal and reproductive health care. However, there is a paucity of evidence of the extent to which these reforms have had impact on the main policy objectives of enhancing service utilization and financial protection. The aim of this systematic review is to assess the existing evidence on the causal impact of health insurance on maternal and reproductive health service utilization and financial protection in low- and lower middle-income countries.

**Methods:**

The review followed the Preferred Reporting Items for Systematic Reviews and Meta-Analyses (PRISMA) guidelines. The search included six databases: Medline, Embase, Web of Science, Cochrane, CINAHL, and Scopus as of 23rd May 2023. The keywords included health insurance, impact, utilisation, financial protection, and maternal and reproductive health. The search was followed by independent title and abstract screening and full text review by two reviewers using the Covidence software. Studies published in English since 2010, which reported on the impact of health insurance on maternal and reproductive health utilisation and or financial protection were included in the review. The ROBINS-I tool was used to assess the quality of the included studies.

**Results:**

A total of 17 studies fulfilled the inclusion criteria. The majority of the studies (82.4%, *n* = 14) were nationally representative. Most studies found that health insurance had a significant positive impact on having at least four antenatal care (ANC) visits, delivery at a health facility and having a delivery assisted by a skilled attendant with average treatment effects ranging from 0.02 to 0.11, 0.03 to 0.34 and 0.03 to 0.23 respectively. There was no evidence that health insurance had increased postnatal care, access to contraception and financial protection for maternal and reproductive health services. Various maternal and reproductive health indicators were reported in studies. ANC had the greatest number of reported indicators (*n* = 10), followed by financial protection (*n* = 6), postnatal care (*n* = 5), and delivery care (*n* = 4). The overall quality of the evidence was moderate based on the risk of bias assessment.

**Conclusion:**

The introduction or expansion of various types of health insurance can be a useful intervention to improve ANC (receiving at least four ANC visits) and delivery care (delivery at health facility and delivery assisted by skilled birth attendant) service utilization in low- and lower-middle-income countries. Implementation of health insurance could enable countries’ progress towards UHC and reduce maternal mortality. However, more research using rigorous impact evaluation methods is needed to investigate the causal impact of health insurance coverage on postnatal care utilization, contraceptive use and financial protection both in the general population and by socioeconomic status.

**Trial registration:**

This study was registered with Prospero (CRD42021285776).

**Supplementary Information:**

The online version contains supplementary material available at 10.1186/s12913-024-10815-5.

## Introduction

Low- and middle-income countries (LMICs) have committed to making progress towards universal health coverage (UHC) as part of the Sustainable Development Goals (SDGs). UHC has been defined by the World Health Organization (WHO) as a state where all people and communities receive the quality health services they need, when they need them, without experiencing financial hardship due to health care costs [1]. Generally, high income countries have attained high levels of service coverage (UHC service coverage index of at least 80 out of 100), however a majority of low- and lower-middle income countries (LLMICs) are still lagging behind (UHC service coverage index of less than 60 out of 100) as of 2022 [2]. The health service coverage index is the average coverage of essential services based on 14 tracer indicators of health service coverage (encompassing reproductive, maternal, newborn and child health, infectious diseases, non-communicable diseases and service capacity and access) among the general and the most disadvantaged population [3, 4]. Similarly, while efforts have been made to decrease catastrophic health expenditure globally, LLMICs continue to face the greatest burden of people being thrust into extreme poverty (spending less than international dollars 1.9 per day) due to out of pocket payments (OOP) on healthcare [5].

In order to advance towards UHC, several countries especially LLMICs, are planning or implementing health financing reforms with a view to introduce or expand some form of health insurance (i.e. prepayment and pooling of funds). Countries that have opted for health insurance schemes – specifically social health insurance (SHI) – have seen an increment in their health expenditure compared to those that have a tax-based model of financing [6, 7]. However, the choice of health financing mechanism does not necessarily have a clear effect on health outcomes (such as increased immunization coverage, reduced under-five mortality) or financial protection [7]. For example, Wagstaff who looked at Organization for Economic Co-operation and Development (OECD) countries found that neither a tax-funded health system nor a SHI system had a significant effect on health outcomes [6] while Gabani et al. who looked at over 124 countries found that transitions from predominantly OOP financing to tax-funded health systems yielded significantly better health outcomes than transitions from predominantly OOP financing to health insurance [7].

An increasing number of LLMICs have started implementing, or are planning to implement health insurance reforms to advance UHC [8–10]. Health insurance can go by different names including SHI, publicly funded health insurance (PFHI), community-based health insurance (CBHI) and private for-profit health insurance based on the pre-payment arrangement within an insurance scheme [11]. The intention, however, is the same for all health insurance systems (especially not for profit health insurance), which is to pool the risk of high-cost health care across a large number of people in order to protect individuals from high unexpected medical costs. Through a system of prepayment for guaranteed access to a predetermined package of health benefits, individuals can benefit from more predictable health care expenses and be protected from catastrophic health expenditure. A number of countries are opting for SHI. SHI refers to a health insurance system where contributions in form of premiums are collected from employees, employers and or government and pooled into an insurance fund [12]. Over time, SHI has been defined to mean insurance schemes where employees and employers both contribute premiums to the insurance fund. In instances where contributions/premiums are paid by government, such insurance has been referred to as PFHI for example in India [13, 14]. PFHI has been implemented in some LLMIC settings, where there is a large informal sector, and inability to pay or collect premiums. In some cases, a health insurance scheme can be a combination of tiered contributions by members and subsidies from the government for example contributory and non-contributory.

A core component of UHC is maternal and reproductive health services (MRH), which has received a lot of attention in the past few decades. It was central to the Millennium Development Goals, specifically Goal 5 aimed at improving maternal health [15]; and it is currently well stipulated within the SDGs. MRH is one of the four categories measured for the UHC service coverage index. The other health services areas under the index are infectious diseases, non-communicable diseases and service capacity and access [16]. Several interventions have been implemented to improve MRH, including sexual and reproductive health and rights interventions. These endeavors have led to the improvement of MRH globally [17]. However, several LLMICs continue to face high maternal mortality ratios (accounting for 94% of all maternal deaths globally) [18], which is far from achieving the target of reducing maternal mortality to 70 deaths per 100,000 live births. Furthermore, women have continued to experience financial barriers when seeking healthcare, and they are found to be more vulnerable to facing financial hardships when accessing care, compared to men [19].

Despite the increasing interest surrounding health insurance, our understanding of the actual causal impact of the implemented reforms remains limited. Several reviews have examined the existing evidence on the impact of health insurance on service utilization and financial protection, but the results are inconclusive [20–23]. A review by Comfort et al. [24] analyzed the effects of health insurance on maternal health services in LMICs. Insurance (a mix of different types of insurance) was found to be consistently associated with increased utilization of facility-based child delivery and delivery assisted by a skilled health worker.

However, Comfort et al.’s study did not address the impact of health insurance on financial protection. In addition, the study examined various types of insurance schemes, including a mix of both for-profit and not-for-profit models. The study also included conditional cash transfers (or CCTs, a kind of demand-side financing). Therefore, based on Comfort et al.'s findings, it can be challenging to discern the specific impact of individual types of insurance. Our study differs from that of Comfort et al. as we specifically focus on well-specified not-for-profit health insurance as the intervention in LLMICs. Furthermore, we have also examined and reported on the impact of insurance on the financial protection of women of reproductive age. Our review constitutes a contribution to the current evidence base on this topic as no previous review has specifically examined the impact of not-for-profit health insurance on maternal and reproductive service utilization and financial protection in LLMICs, despite the recognition that MRH is among the four core categories of essential health services under UHC [4].

Our study aims to review the existing evidence of the causal impact of health insurance on maternal and reproductive service utilization and financial protection in LLMICs to inform ongoing health financing reform discussions and identify evidence gaps for future research.

## Methods

The systematic review followed the Preferred Reporting Items for Systematic Reviews and Meta-Analyses (PRISMA) guidelines [25]. In addition, we used the PICO framework [26] to guide the study scope. The study is registered with PROSPERO, registration number CRD42021285776. We searched electronic databases to identify published articles, and bibliographies of included articles were screened to identify missed articles that fulfilled the inclusion criteria.

To narrow the scope of our study, we employed the PICO framework with the following parameters – Population: Women of reproductive age residing in low- and lower middle-income countries as classified by the World Bank [27] as of 1st July 2021 – Intervention: health insurance – Comparator: uninsured women – Outcomes: two types of outcomes were considered, i) utilisation of maternal and reproductive health services, and ii) financial protection. Outcomes on utilisation of MRH included contraceptive use, number of antenatal care (ANC) visits, delivery at health facility. Outcomes on financial protection included catastrophic health expenditure and impoverishment impact of out-of-pocket expenditure (OOPE).”

### Studies reporting impact

In this study, we reviewed studies that reported impact, i.e. studies that employed a research design enabling the identification of a causal effect of the intervention on an outcome of relevance. We defined studies that report impact as studies that estimate the causal influence the intervention (health insurance) has on a given outcome (MRH and financial protection indicators).

### Intervention

The intervention in this study is any not-for-profit health insurance. Health insurance is the protection of registered members (beneficiaries) from high costs of/expenditure on health services by pooling resources through payment of agreed periodic premiums. A person or entity (employer or government) pays a periodic premium to enable them access to health services without requiring them to pay for the services. In some cases where there is a co-payment, the beneficiary pays a small amount or portion of the cost of services they receive. This arrangement aims at sharing the financial risks associated with falling ill and needing medical care.

### Inclusion criteria

Our review applied the following inclusion criteria. We included articles that:

- Reported on any specific type of not-for-profit health insurance. The reason was to focus on health insurance implemented with the intention of progressing towards UHC.
- Reported on the impact of health insurance on MRH service utilization and/or financial protection of people accessing MRH services, and where any MRH service is included in the health benefit package of the insurance scheme. MRH in this study covers contraception, pre- and postnatal services, and delivery care.
- Used experimental and/or quasi experimental study designs.
- Were published from 2010 onwards and in English. We included studies from 2010 to capture the most recent evidence, as insurance schemes undergo reforms over time with likely implications on their respective performance. Relevant unpublished studies in the form of reports were also considered.

### Exclusion criteria

Articles were excluded if they:

- Reported on the impact of private for-profit insurance only or aggregated all types of insurance (both for-profit and not-for-profit) as one. Excluding such articles was necessary because grouping different insurance types as a single intervention makes it challenging to differentiate the specific impact of each insurance type.
- Adopted a non-experimental study design prohibiting the identification of a causal effect.
- Reported on the impact of health insurance qualitatively.
- Were published in languages other than English.
- Had unavailable full texts.

### Databases searched

We searched for published literature in selected electronic databases and bibliographies. Databases included Medline (PubMed), Embase, Web of Science, CINAHL, Cochrane and Scopus. Additionally, we reviewed bibliographies of included articles to find other relevant articles that might have been missed in the search. It should be noted that although no time restrictions were included in the search strategy, studies published before 2010 were excluded at the screening stage.

### Search strategy

The six electronic databases were searched on the 31st of October 2021, with an update of the search carried out on 23rd May 2023. The search strategy included all keywords and respective keyword variations for the five keyword domains: health insurance, impact, utilisation, financial protection, and maternal and reproductive health. Search strategies were customised for the respective databases (Supplementary Material 1: Appendix 1).

### Study selection

The PRISMA guidelines [28] were followed in the articles screening and selection process. The articles retrieved from the search were uploaded to Covidence systematic review software [29], where duplicates were removed. Initially, at least two independent researchers (PT, SPK, and JK) carried out screening for each title and abstract. Subsequently, full text screening was conducted by PT and JK, following a standard protocol. In the event of any conflict, a fourth researcher (JS) was available to review the conflict and make the final decision.

### Data extraction

We developed a data extraction template in Microsoft Excel, which was piloted on ten randomly selected articles and necessary adjustments were made. We extracted data on author, year of publication, target group, study design, country, geographic location, setting (rural/urban/mixed), level of health facility, study participants, type of insurance, year of implementation of insurance, source of data, year of data collection, analysis methods used, description of the insurance, type of membership (voluntary/compulsory), enrolment requirements, services covered by insurance, services received, insurance coverage, premium, reimbursement rates, co-payments, OOPE, indicators used in measuring utilisation, financial protection and their definitions, proportion of households experiencing catastrophic heath expenditure (CHE), measures used for impact, theoretical framework used, reported impact (adjusted and unadjusted), and correction of self-selection among others.

### Quality assessment and risk of bias in individual studies

The quality of the evidence was assessed through a two-step process, including: 1) using a tool for assessing the risk of bias in each study and 2) using the GRADE criteria to determine the level of certainty of the evidence.

The study design of an article being assessed determined the quality assessment tool to be used. Since we did not find any randomised studies, we resorted to a tool suitable for non-randomised studies. We used the Risk of Bias in Non-randomised Studies of Interventions (ROBINS-I) tool developed by the Development and Evaluation (GRADE) working group [30]. The tool rates the risk of bias in seven domains, 1) Bias due to confounding, 2) Bias in selection of participants into the study, 3) Bias in classification of interventions, 4) Bias due to departures from intended interventions, 5) Bias due to missing data, 6) Bias in measurement of outcomes, and 7) Bias in selection of reported results [31]. The study can be rated as low risk of bias, moderate risk of bias, or serious risk of bias based on the respective guiding questions in the tool for each domain. The overall extent of bias of a study is determined by the respective domain ratings, following the algorithm in the guide. The above tool was selected following the findings of a systematic review by Ma et al [32], describing the ROBINS-I tool as one of the most reliable tools available for quality assessment.

The certainty level of evidence of each study was then determined following the GRADE criteria based on the ROBINS-I tool [33]. It involved three steps: 1) establishing the initial level of certainty as advised by GRADE, 2) considering lowering or raising the level of certainty and 3) determining the final certainty rating. The certainty level of the evidence could be high, moderate, low, or very low depending on the rating. A non-randomised study’s evidence is initially rated as high level of certainty, which is then downgraded by a level or two depending on how it performs in the following domains: limitations in the detailed study design and execution; inconsistency (or heterogeneity); indirectness (PICO and applicability); imprecision; and publication bias. The downgrade of the certainty level of the evidence is mitigated (increased) by the magnitude of the effect estimates.

### Reporting, summary measures and synthesis of results

The reporting was both descriptive and analytical. For extracted quantitative data, we reported summary measures. Indicators reported by the different studies were categorised into MRH service utilisation indicators and financial protection indicators with several subcategories each. Additionally, we elicited the covariates used in the adjustment of results from the studies and categorised these into characteristics including mother’s demographic, households, partners, communities, and mother’s perceptions. The reported impact of the insurance on MRH service utilisation and financial protection was summarised in five groups: positive and significant impact, positive and not significant impact, no impact, negative and not significant impact, and negative and significant impact. The impact findings were summarised in a table showing the proportion of studies that reported a positive significant impact for each respective indicator. Studies that had a high risk of bias were excluded in the calculation of the proportions as shown in Table 3.

## Results

**Fig. 1:**
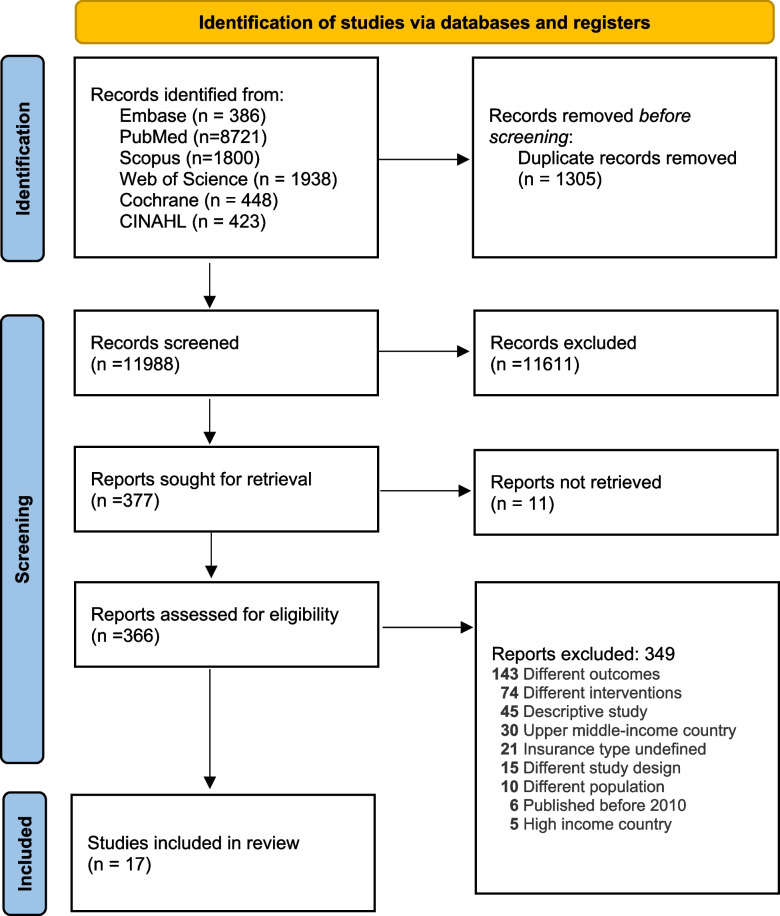
PRISMA flow chart

The systematic literature search yielded a total of 11,988 studies after deduplication. Following title and abstract screening and full text review, we included 17 studies that fulfilled our eligibility criteria. Figure 1 shows the flow of selection process including reasons for exclusion of articles at the full text reading stage.

## Characteristics of included studies

Table 1 provides an overview of key characteristics of the included studies.

**Table 1:** Study characteristics of included studies

S/N	Study	Study design	Country	Geographical location	Setting (Rural/ urban)	Target group/study population	Type of insurance	Source of data
1	Samarakoon et al. 2020 [34]	Quasi experimental	Indonesia	National	Both	Women aged 15–45	Social HI	IFLS 2000 and 2007
2	Agbanyo et al. 2021 [35]	Quasi experimental	Ghana	National	Both	Women aged 15–49 that had delivered in the last 5 years	Social HI	DHS 2008 & 2014
3	Ravit et al. 2020 [36]	Quasi experimental	Mauritania	National	Both	Women aged 15–49 that had delivered in the last 2 years	CBHI	MICS 2015
4	Chang et al. 2018 [37]	Quasi experimental	Rwanda	National	Both	Women aged 15–49 that had delivered in the last 5 years	CBHI	DHS 2005, 2008, 2010
5	Rashad et al. 2019 [38]	Quasi experimental	Egypt	National	Both	Women aged 15–49 that had delivered in the last 5 years	Social HI	DHS 2014
6	Gouda et al. 2016 [39]	Quasi experimental	Philippines	National	Both	Women aged 15–49 that had delivered in the last 5 years	Social HI	DHS 2014
7	Philibert et al. 2017 [40]	Quasi experimental	Mauritania	National	Both	Women aged 15–49 that had delivered in the last 5 years	CBHI	DHS 2001, NSIMM 2003 & MICS 2007, 2011
8	Anindya et al. 2020 [41]	Quasi experimental	Indonesia	National	Both	Women aged 15–49 that had delivered in the last 5 years	Social HI	DHS 2017, 2012
9	Kuwawenaruwa et al. 2019 [42]	Quasi experimental	Tanzania	Regional	Rural	Women that had delivered in the last 12 months	Social HI	Survey
10	Aizawa 2019 [43]	Quasi experimental	Indonesia	National	Both	Women aged 15–49 that had delivered in the last 5 years	Social HI	IFLS-6
11	Bonfrer et al. 2016 [44]	Quasi experimental	Ghana	National	Both	Women aged 15–49 that had delivered in the last 5 years	Social HI	DHS 2008
12	El Omari et al. 2021 [45]	Quasi experimental	Philippines	National	Both	Indigent Women aged 15–49 that had delivered in the last 2 years	Social HI	DHS 2013
13	Wang et al. 2017 [46]	Quasi experimental	Ghana, Indonesia & Rwanda	National	Both	Women aged 15–49 that had delivered in the last 5 years	Social HI	GDHS 2008, IDHS 2012 & RDHS 2010
14	Kofinti et al. 2022 [47]	Quasi experimental	Ghana	National	Both	Women aged 15–49 that had delivered in the last 5 years	Social HI	DHS 2014
15	Bousmah et al. 2022 [48]	Quasi experimental	Senegal	Regional	Rural	Women aged 15–49 that had delivered in the last 2 years	CBHI	Survey
16	Mussa et al. 2023 [49]	Quasi experimental	Ethiopia	Regional	Rural	Women of reproductive age	CBHI	Survey
17	Garg et al. 2023 [50]	Quasi experimental	India	National	Both	Women having a delivery in the last one year	PFHI	IFLS-5

*HI* Health insurance, *CBHI* Community based health insurance, *IFLS n* Indonesia family living standards survey (*n* stands for the round), *NSIMM* National survey on infant mortality and malaria, *GDHS* Ghana demographic and health survey, *IDHS* Indonesia demographic and health survey, *RDHS* Rwanda demographic and health survey, *MICS* Multiple cluster survey, *DHS* Demographic and health survey

All the included studies were quasi experimental studies (*n* = 17). Quasi experimental studies are non-randomised studies that evaluate an intervention with the aim of demonstrating causality between the intervention and outcome [51]. Ghana and Indonesia had the most studies with three each. These were followed by the Philippines (*n* = 2), and Mauritania (*n* = 2). The review included one study from each of the following countries: Tanzania, Egypt, Rwanda, Ethiopia, India, and Senegal (Table 1). Additionally, there was a multi-country study that encompassed three countries: Ghana, Rwanda, and Indonesia [46]. Most studies (*n* = 14) were nationally representative of the population, while the remaining three were carried out in specific region(s) within the specified country [42, 48, 49]. Three studies were specifically conducted in rural settings [42, 48, 49], and no studies focused on urban settings exclusively. The rest of the studies (*n* = 14) covered both rural and urban areas. All the studies included in the review involved female participants of childbearing age from 15 years old. The majority of the studies (*n* = 13) specifically focused on women aged between 15 and 49. The studies focused on three types of health insurance, including social health insurance (e.g. Ghana), community-based health insurance (e.g. Rwanda), and publicly-funded health insurance (e.g. India). The data sources used were mostly secondary data (*n* = 14), specifically demographic health survey (DHS) data [52], Multiple Indicator Cluster Survey (MICS) and Family Life Survey (FLS).

## Quality assessment of included studies

**Fig. 2:**
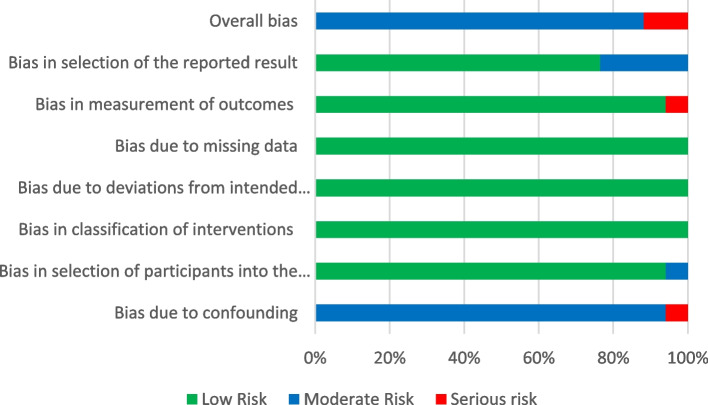
Assessment of the risk of bias of the studies according to the seven domains, using the ROBINS-I tool

The overall quality of assessed studies was rated as moderate. A total of 12 studies were rated as having a high level of certainty of evidence [36–39, 41, 43–46, 48–50], two studies had moderate while those rated low and very low were two [40, 42] and one [34], respectively. This assessment was based on the categorization of the risk of bias using the ROBINS-I tool. Overall, the majority of the studies (*n* = 15; 88.2%) were categorised as having moderate risk of bias, and two studies were rated as having serious risk [34, 42]. No study was found with an overall low risk of bias. All studies were rated as low risk of bias in three domains: bias in classification of interventions, bias due to departures from intended interventions, and bias due to missing data as shown in Fig. 2. For the domain of bias in selection of reported results, the majority of the studies (*n* = 16, 94.1%) were rated as low risk of bias, while the rest (*n* = 1, 5.9%) was rated as moderate risk [40]. One study showed serious risk of bias due to confounding [34], and one [42] study was assessed to have serious risk of bias in the measurement of outcomes. The table with the assessment results is included in Supplementary Material 1: Appendix 2, and Fig. 2 shows the ratings by domain as well as the overall rating of bias.

## Indicators used to measure the impact

Table 2 shows the indicators used to measure the impact of health insurance on MRH service utilisation and financial protection. There was a large variation in the number of indicators per category of MRH services, and the frequency to which they were reported in the studies. Regarding the number of indicators per category, ANC had the greatest number of reported indicators (*n* = 10), followed by financial protection (*n* = 6), postnatal care (*n* = 5), and delivery care (*n* = 4). Contraception had only one indicator, with three studies reporting on this indicator [34, 40, 42]. Regarding the frequency of use of the indicators, under the ANC category, the most common indicator was having at least four ANC visits during pregnancy (*n* = 9, 52.9%), which was also the second most reported MRH indicator in this review. For delivery care, delivery at a health facility was the most reported indicator in the delivery care category (and the most reported indicator in this review) (*n* = 14, 82.4%); followed by delivery by skilled attendant (*n* = 7, 41.2%). For the postnatal care category, having postnatal care (without specifying the point or time of access) was the most common indicator used in this category (*n* = 4, 23.5%) [38, 40, 41, 45]. For financial protection, six indicators were reported. OOPE on delivery services was reported in four studies (23.5%) [34, 42, 43, 50]. OOPE due to ANC, OOPE due to C-section, financial distress after C-section, and CHE were each reported in one study. Financial distress was defined by Garg et al. as a situation where a patient, or their household member, borrowed money or sold their assets to cover the OOPE due to seeking maternal health care [50].

**Table 2:** Indicators used to measure impact on utilisation of maternal and reproductive health services and financial protection

Objective	Category of indicator	Indicator subcategory	Indicator	Studies (*N* = 17)	
				**Total (*n*)**	**Proportion (%)**
**Utilisation**	Contraception	Contraceptive use	Contraceptive use	3	17.6
	ANC	Timing of ANC	Having first ANC visit in first trimester	1	5.9
			Time to first ANC visit	1	5.9
		Frequency of ANC visits	At least 4 ANC visits	9	52.9
			Number of ANC visits during a pregnancy	2	11.8
			Number of ANC visits in 1st trimester	2	11.8
			Number of ANC visits in 2nd trimester	1	5.9
			Number of ANC visits in 3rd trimester	1	5.9
		Place of ANC visit	ANC at health facility	1	5.9
		Skilled staff during ANC visit	ANC with skilled staff	3	17.6
		Components of ANC visits	Complete assessment in ANC	1	5.9
	Delivery	Place of delivery	Delivery at health facility	14	82.4
		Skilled staff during delivery	Delivery assisted by skilled attendant	7	41.2
		Type of delivery	C section delivery	3	17.6
		Safety of delivery	Maternal near miss	1	5.9
	Postnatal care	Postnatal care attendance	Postnatal care	4	23.5
		Timing of postnatal care	PNC before leaving facility	1	5.9
			PNC after discharge	1	5.9
			Postnatal care at health facility < 2 month	1	5.9
		Skilled staff during postnatal care	PNC with skilled provider	1	5.9
**Financial protection**	Cost of services to the patient	OOPE due to ANC	OOPE due to ANC	1	5.9
		OOPE due to delivery	OOPE due to delivery	4	23.5
			OOPE due to C-section	1	5.9
		OOPE due to PNC	OOPE due to PNC	1	5.9
		Financial hardship	Financial distress after c-section	1	5.9
			CHE	1	5.9

## Impact of health insurance on MRH service utilisation and financial protection

Studies reported a positive effect of the health insurances on ANC and delivery care indicators, with a clear significant positive impact reported for the most used indicators (having at least four ANC visits, delivery at a health facility, and delivery assisted by a skilled attendant) as shown in Table 3. Specifically, 85.7% of the studies found a significant positive impact between health insurance and delivery with assistance from a skilled attendant, 83.3% reported a significant positive impact on delivery at a health facility, and 75.0% indicated a significant positive impact on having at least four ANC visits during pregnancy. In contrast, the evidence on the impact of health insurance on contraceptive use [40], postnatal care [36, 38, 40, 41, 45] and financial protection [43, 48, 50] indicators was scanty, variable and inconclusive.

**Table 3:** Number and proportion of studies reporting significant positive impact of health insurance on maternal and reproductive health service utilisation and financial protection indicators

Objective	Category of indicator	Indicator subcategory	Indicator	Studies		
				Significant positive (*n*)	Total studies (*N*)	Proportion (%)
Utilisation	Contraception	Contraceptive use	Contraceptive use^b^	0	1	0.0
	ANC	Timing of ANC	Having first ANC visit in first trimester	1	1	100.0
			Reduction in time to first ANC visit	1	1	100.0
		Frequency of ANC visits	At least 4 ANC visits^a^	6	8	75.0
			Number of ANC visits during a pregnancy	1	2	50.0
			Number of ANC visits in 1st trimester	1	2	50.0
			Number of ANC visits in 2nd trimester	1	1	100.0
			Number of ANC visits in 3rd trimester	1	1	100.0
		Skilled staff during ANC visit	ANC with skilled staff	1	3	33.3
		Components of ANC visits	Complete assessment in ANC	1	1	100.0
	Delivery	Place of delivery	Delivery at health facility^b^	10	12	83.3
		Skilled staff during delivery	Delivery assisted by skilled attendant	6	7	85.7
		Type of delivery	Reduction in C section delivery	2	3	66.7
	Postnatal care	Postnatal care attendance	Postnatal care	2	4	50.0
		Timing of postnatal care	PNC before leaving facility	0	1	0.0
			PNC after discharge	0	1	0.0
		Skilled staff during postnatal care	PNC with skilled provider	1	1	100.0
Financial protection	Cost of services to the patient	OOPE due to delivery	Reduction of OOPE due to delivery^b^	1	2	50.0
			Reduction of OOPE due to C-section	0	1	0.0
		Financial hardship	Reduction of financial distress after c-section	1	1	100.0
			Reduction of CHE	0	1	0.0

Level of significance of the impact measure reported in this table is 95% Confidence interval

Studies rated as having serious risk of bias were excluded from this table

*ANC* Antenatal care, *CHE* Catastrophic health expenditure, *OOPE* Out of pocket expenditure, *PNC* Postnatal care

^a^One study was excluded from the calculation because of having serious risk of bias

^b^Two studies were excluded from the calculation because of having serious risk of bias

Certain indicators (ANC at health facility, postnatal care visit at health facility in less than 2 months after delivery, OOPE due to ANC, and OOPE due to PNC) were not included in the analysis, because these indicators were only reported in articles that were excluded due to their serious risk of bias.

Table 4 shows the magnitude of the impact reported by each study for indicators that were reported by more than one study.

**Table 4:** Magnitude of impact by study

Authors	country	Sample size	Measure	Effect size	Lower bound	Upper bound	Standard error	Standard deviation	Significant at 95% CI
**At least 4 ANC visits**									
** Ravit et al. 2020 **[36]	Mauritania	1496	ATE	0.11	0.06	0.16			Significant
** Rashad et al. 2019 **[38]	Egypt	9960	ATT	0.041			0.01		Significant
** Philibert et al. 2017 **[40]	Mauritania	3520	Absolute risk	0	-0.05	0.05			Not Significant
** Anindya et al. 2020 **[41]	Indonesia	5705	ATT	0.074	0.048	0.099			Significant
** Bonfrer et al. 2016 **[44]	Ghana	2002	ATT	0.07					Significant
** El Omari et al. 2021 **[45]	Philippines	3648	ATE	0.085				0.109	Significant
** Wang et al. 2017 **[46]	Ghana	1753	ATT	0.0771			0.0257		Significant
** Wang et al. 2017 **[46]	Indonesia	14,318	ATT	0.026			0.006		Significant
** Wang et al. 2017 **[46]	Rwanda	6016	ATT	0.0195			0.0203		Significant
** Mussa et al. 2023 **[49]	Ethiopia	1564	ATE	0.004	-0.056	0.063			Not Significant
**Number of ANC visits during a pregnancy**									
** Kofinti et al. 2022 **[47]	Ghana	4169	ATE	0.432			0.101		Significant
** Bousmah et al. 2022 **[48]	Senegal (contributory)	804	ATE	0.565			0.18		Significant
** Bousmah et al. 2022 **[48]	Senegal (non-contributory)	983	ATE	− 0.325			0.36		Not significant
**Number of ANC visits in 1st trimester**									
** Aizawa 2019 **[43]	Indonesia (non-contributory insurance)	3717	ATE	–0.0823			0.0597		Not significant
** Aizawa 2019 **[43]	Indonesia (contributory insurance)	3314	ATE	0.0721			0.0717		Not significant
** Wang et al. 2017 **[46]	Ghana	1753	ATT	0.0184			0.0365		Not significant
** Wang et al. 2017 **[46]	Indonesia	14,318	ATT	0.017			0.008		Significant
** Wang et al. 2017 **[46]	Rwanda	6016	ATT	0.017			0.0206		Not significant
**ANC with skilled staff**									
** Philibert et al. 2017 **[40]	Mauritania	4029	Absolute risk	0.06	0.01	0.11			Significant
** Bonfrer et al. 2016 **[44]	Ghana	2002	ATT	0.05					Not significant
** Mussa et al. 2023 **[49]	Ethiopia	1564	ATE	0.017	-0.053	0.087			Not significant
**Delivery at health facility**									
** Agbanyo et al. 2021 **[35]	Ghana	8818	Marginal effects	0.203			19.56		Significant
** Ravit et al. 2020 **[36]	Mauritania	2602	ATE (District hospital)	0.04	0	0.08			Significant
** Rashad et al. 2019 **[38]	Egypt	9960	ATT	0.034			0.01		Significant
** Gouda et al. 2016 **[39]	Philippines	1376	ATT	0.0973			0.035		Significant
** Philibert et al. 2017 **[40]	Mauritania		Absolute risk	− 0.04	-0.09	0.13			Not Significant
** Anindya et al. 2020 **[41]	Indonesia	5705	ATT	0.102	0.075	0.127			Significant
** Aizawa 2019 **[43]	Indonesia (non contributory insurance)	3720	ATE	0.203			0.0122		Significant
** Aizawa 2019 **[43]	Indonesia (contributory insurance)	3317	ATE	0.13			0.0125		Significant
** Bonfrer et al. 2016 **[44]	Ghana	2002	ATT	0.12					Significant
** Wang et al. 2017 **[46]	Ghana	1837	ATT	0.1058			0.0319		Significant
** Wang et al. 2017 **[46]	Indonesia	14,954	ATT	0.049			0.009		Significant
** Wang et al. 2017 **[46]	Rwanda	6122	ATT	0.0745			0.0186		Significant
** Kofinti et al. 2022 **[47]	Ghana	4169	ATE	0.062			0.017		Significant
** Bousmah et al. 2022 **[48]	Senegal (contributory)	804	ATE	0.349			0.08		Significant
** Bousmah et al. 2022 **[48]	Senegal (non contributory)	983	ATE	0.238			0.12		Significant
** Mussa et al. 2023 **[49]	Ethiopia	1564	ATE	-0.005	-0.065	0.056			Not Significant
**Delivery assisted by skilled attendant**									
** Ravit et al. 2020 **[36]	Mauritania	2400	ATE	0.08	0.04	0.12			Significant
** Chang et al. 2018 **[37]	Rwanda	1913	Odds Ratio	1.158			0.038		Significant
** Anindya et al. 2020 **[41]	Indonesia	5705	ATT	0.03	0.015	0.045			Significant
** Bonfrer et al. 2016 **[44]	Ghana	2002	ATT	0.1					Significant
** El Omari et al. 2021 **[45]	Philippines	3648	ATE	0.234				0.074	Significant
** Kofinti et al. 2022 **[47]	Ghana	4169	ATE	0.068			0.016		Significant
** Mussa et al. 2023 **[49]	Ethiopia	1564	ATE	-0.008	-0.072	0.056			Not Significant
**C section delivery**									
** Ravit et al. 2020 **[36]	Mauritania	1796	ATE	− 0.01	− 0.05	0.03			Not Significant
** Philibert et al. 2017 **[40]	Mauritania	4029	Absolute risk	− 0.02	-0.04	-0.01			Significant
** Bonfrer et al. 2016 **[44]	Ghana	2002	ATT	0.06			0.016		Significant
**Postnatal care**									
** Rashad et al. 2019 **[38]	Egypt	9960	ATT	0.03			0.016		Not Significant
** Philibert et al. 2017 **[40]	Mauritania	3996	Absolute risk	− 0.01	-0.07	0.05			Not Significant
** Anindya et al. 2020 **[41]	Indonesia	5705	ATT	0.04	0.022	0.057			Significant
** El Omari et al. 2021 **[45]	Philippines	3648	ATE	0.093				0.156	Significant
**Reduction of OOPE due to delivery**									
** Aizawa 2019 **[43]	Indonesia (non-contributory insurance)	3720	ATE	1,136,966 IDR					Significant
** Aizawa 2019 **[43]	Indonesia (contributory insurance)	3317	ATE	676,402 IDR					Significant
** Garg et al. 2023 **[50]	India	33,345	ATE	22.89 INR					Not significant

*ATE* Average treatment effect*, ATT* Average treatment effect on the treated*, IDR* Indonesian rupiah*, INR* Indian rupee

ANC: Health insurance increased the chance of a pregnant woman having at least four ANC visits. The magnitude of the positive significant impact of health insurance on receiving at least four ANC visits during a pregnancy ranged between approximately 2% [46] and 11% [36]. Insurance increased the total number of ANC visits during pregnancy. The magnitude of positive significant impact of health insurance on the number of ANC during pregnancy ranged from 43% [47] to 56% [48]. On the other hand, insurance did not have a significant positive impact on having an ANC visit in the first trimester except for Indonesia [46].

Delivery care: Health insurance increased chances of having a delivery at a health facility and delivery by a skilled attendant. Studies that reported a significant positive impact of health insurance on delivery at a health facility found a magnitude ranging from approximately 3% [38] to 34% [48]. The magnitude of the impact ranged from 3% [41] to 23% [45] for having a delivery assisted by a skilled attendant.

Postnatal care: Health insurance showed an increase in the chance of receiving postnatal care but only 50% of the studies reporting on the impact of health insurance on postnatal reported a significant positive increase. The magnitude of the health insurance on postnatal care among studies that reported positive significant impact was 4% [41] and 9% [45].

Reduction of OOPE: Evidence suggests that health insurance has generally reduced OOP payments for MRH services. However, of the two studies that reported on OOPE only one found a significant reduction in OOPE of 1,136,966 Indonesian Rupiah (IDR) and 676,402 IDR for non-contributory and contributory health insurance in Indonesia respectively [43].

## Methods used to estimate the impact of health insurance on MRH service utilisation

No study used randomisation in allocating participants to the intervention or control groups.

A wide range of statistical methods were applied in the studies (Table 5). Propensity Score Analysis/Matching (PSM) was the most used statistical methods (58.8%), followed by difference-in-difference (DID) analysis (11.8%). Some studies utilised more than one method; for example, Samarakoon et al [34] used both PSM and DID. The effect measures used were mostly Average Treatment Effect (ATE) (47.1%), and Average Treatment Effect on the Treated (ATT) (35.3%).

**Table 5:** Data, methods and covariates used in studies that reported adjusted results

*Methods*	Studies (*N* = 17)	
	***n***	**%**
**Data sources used**		
Publicly available datasets e.g., DHS, MICs	14	82.4
Study specific surveys	3	17.6
**Statistical methods used**		
Propensity Score Analysis/Propensity Score Matching	10	58.8
Difference In Difference	2	11.8
Regression discontinuity design	1	5.9
Others	4	23.5
**Effect measures**		
Average Treatment Effect	8	47.1
Average Treatment Effect on the Treated	6	35.3
Marginal effects	1	5.9
Odds ratios	1	5.9
Absolute risk difference	1	5.9
**Self-selection adjustment**		
Yes	17	100.0
**Reference group**		
Uninsured/ before insurance	17	100.0
***Covariates***		
**Demographic characteristics of the woman**^a^		
Education	13	76.5
Age	13	76.5
Marital status	10	58.8
Employment status	8	47.1
Parity/number of children	6	35.3
Religion	5	29.4
Ethnicity	2	11.8
**Household characteristics**^a^		
Wealth status	13	76.5
Exposure to media	6	35.3
Education status of the household head	6	35.3
Household size	5	29.4
Age of household head	2	11.8
Sex of household head	2	11.8
**Partner demographics**		
Partner's employment status	1	5.9
Partner education	1	5.9
**Community characteristics**^a^		
Place of residence (rural/urban)	10	58.8
Geographical location	5	29.4
Distance to the health facility	3	17.6
**Perceptions**		
Distance to health facility perceived as a difficulty	1	5.9
Quality of care	1	5.9
Insurance coverage	1	5.9

^a^Only methods used by more than one study were included in this table

Several methods were used to adjust for self-selection, such as PSM, DID, conditional mixed process framework (CMP) (e.g. Agbanyo et al [35]), entropy balance weighting of observed characteristics (e.g. Aizawa [43]) and coarsened exact matching (CEM) methods (e.g. Chang et al [37]). Anindya et al [41] used more than one method, specifically PSM followed by CEM for sensitivity analysis and robustness check.

### Covariates adjusted for in the studies

Table 5 presents the covariates that were adjusted for in the studies. Overall, the most used covariates were age, the education level of the woman, and wealth status of the household; with each being used in 76.5% of all studies. This was followed by place of residence (rural/urban) and marital status, with each at 58.8%. The other covariates were used in less than 50% of the studies that adjusted for covariates.

## Discussion

Our review shows that there is considerable evidence on the impact of health insurance on ANC and delivery care service utilisation. However, there is a scarcity of evidence on the impact of health insurance on the financial protection of women seeking MRH services, utilisation of postnatal care, and contraception. We found that health insurance has a significant positive impact on ANC and delivery care service utilisation specifically having at least four ANC visits, delivery at a health facility and having a delivery assisted by a skilled attendant. However, findings regarding its impact on financial protection, contraception, and postnatal care were inconclusive.

### ANC and delivery care utilisation

Among the articles reviewed, recent evidence shows that health insurance generally exhibits a positive impact on ANC and delivery care service utilisation. This is in line with the findings of Spaan et al. and Erlangga et al. reporting that social health insurance and CBHI improved general health service utilisation [20, 21]. With comparison to Acharya et al [22] – who reported inconclusive results on the impact of health insurance on general health service utilisation among the informal sector – the evidence that was reported on MRH service utilisation in that study concurs with our findings.

On the other hand, our findings differ from Comfort et al., who stated that there was no evidence that insurance increased maternal health service utilisation [24]. The statement was premised on the fact that Comfort et al. did not identify any studies that used randomised methods. Comfort et al. argued that causality could not be established without randomisation of the intervention. However, quasi experimental studies can estimate causation which are the only studies we included in our review. In addition, as shown in Table 1, all the studies included in our review were published after the publication of Comfort et al.’s review (2013). This indicates that studies which estimated the causal relationship between health insurance and MRH are recent.

For countries that are still experiencing high MMR [53, 54], the evidence available on the positive impact of health insurance on at least four ANC visits, delivery at a health facility and having a delivery assisted by a skilled attendant can inform the country’s health financing reforms, encourage implementation, and expansion of such insurance schemes as an intervention to increase access to care and reduce MMR. MRH services such as attending ANC and having a health facility-based delivery have been highlighted as some of the ways to counter occurrence of maternal mortality[54], and investment in these services was found to be cost-effective [17, 55].

### Limited evidence on financial protection when accessing MRH

The available evidence suggests that health insurance plays a role in reducing OOPE. However, it is important to note that the evidence in this area is weak, with only a limited number of studies reporting on OOPE indicators. The findings are variable and inconclusive, particularly regarding the likelihood of CHE and the reduction of OOPE specifically related to delivery care. This finding contrasts with the results of a previous systematic review examining financial protection in a broader context [20]. Health insurance is known to reduce CHE generally. However, we did not find any evidence of a positive impact of health insurance in reducing CHE in the MRH context. It should be noted that this review found very few studies (less than five) that investigated the impact of health insurance on the financial protection of women seeking MRH services in LLMICs. Globally, LMICs bear the highest proportion of OOPE on health. OOPE on health was 43.21% of the total in low-income countries, and 48.17% for lower middle-income countries; meanwhile, the global average is at 18.01% based on the World Bank estimates of 2019 [56]. Countries that channel larger shares of total health expenditure through prepayment schemes such as health insurance tend to have lower levels of OOPE. As an example, in 2019, the level of OOPE as a proportion of current health expenditure in Indonesia was 34.76%, while in Ghana it was 36.22% which is lower than to the LMIC average. The OOP costs to the patient are found to increase with the increasing level of care. For example in Vietnam, community health facilities had a lower cost for deliveries compared to district and higher-level hospitals [57]. Health insurance could be key in protecting populations from financial hardship, although, more evidence is necessary to see whether there is substantive impact of health insurance on the financial protection of mothers or women seeking MRH services, especially among the different wealth quintiles, underserved and vulnerable groups of the population.

### Inconclusive results on contraception and postnatal care utilisation

The evidence on the impact of health insurance on contraception and postnatal care service utilisation was scarce and inconclusive. Specifically, there was very little evidence on the impact of health insurance on the use of contraception. These findings differ from that of Comfort et al., who found a positive association between health insurance and postnatal care utilisation [24]. The difference in findings between our study and that of Comfort et al. could potentially be attributed to their inclusion of cross-sectional studies with less rigorous methods.

For contraceptive use, the inconclusive results could be partly explained by the limited insurance coverage for contraceptives in some countries, where the reimbursable contraceptive options are few. Moreover, the reimbursable contraceptive options may not be the most preferred by the society. For example, Ghana has just officially included long-term contraceptive options (such as permanent methods, intrauterine devices (IUDs), implants, and injectables) in the National Health Insurance Scheme benefit package [58]. On the other hand, cultural, social, and normative practices surrounding postnatal care, as well as the lack of awareness of the clinical postnatal care guidelines may partly explain the inconclusive evidence on the use of postnatal care [59, 60]. In addition, despite the importance of postnatal care and contraceptive use in reducing maternal mortality [18], few studies have evaluated indicators in these areas and the quality of studies examining contraceptive use was moderate to low. The finding regarding the scarcity of evidence on postnatal care in LMICs is not unique to this study, as it has been reported in recent research as well [61]. Further research is needed to better understand the impact of health insurance on postnatal care and contraceptive utilisation.

### Indicators used to measure MRH and the mismatch with international recommendations

Most of the indicators used to measure MRH service utilisation were related to ANC. This may be in part due to the well-established evidence regarding the positive effect of ANC on maternal health related outcomes. Moreover, this aligns with the long standing WHO ANC model (sometimes called basic or focused ANC) introduced in the 1990s, which recommended that a pregnant woman should have at least four ANC visits/contacts during pregnancy [62, 63]. However, WHO recently updated their recommendations, increasing the number of ANC visits/contacts to eight [64]. Unfortunately, our review did not identify any articles that specifically used at least eight visits as an indicator for ANC.

For postnatal care, WHO recommended a minimum of four postnatal care contacts for mothers. These recommended contacts include the first contact within 24 h after delivery, the second contact between 48 and 72 h, the third contact between seven and 14 days, and the fourth contact in the sixth week after delivery [65, 66]. However, there was a mismatch between the WHO recommended indicators and the indicators reported in these studies. This indicates that more publicity/sensitization on this important component of the MRH service delivery spectrum is vital. Authors should be encouraged to use recommended indicators to measure the impact of an intervention (health insurance) towards the achievement of global targets and allow for comparison across countries.

### Methods used by studies

Propensity score matching was the most popular method used in studies. This conforms to the assertion of Abadie and Cattaneo (2018) that noted an increasing use of matching techniques by researcher partly because of the flexibility of the methods and the failure of ordinary linear regression to estimate conventional treatment effect parameters like ATE and ATET [67]. In addition, matching makes it possible to estimate treatment effects in the absence of experimental data in evaluation research [68].Despite the importance of propensity score matching in determining causal inference, it relies on the assumption of conditional independence which may not hold in some instances especially when there are unobservable variables that influence both the treatment and outcome [68].

Different covariates were used to construct statistical models. Some authors selected covariates based on variable significance level, while others based their selection on the confounding relationship between the exposure and outcome. To have evidence of high certainty, it is necessary to adjust the results based on confounders which can be identified using the directed acyclic graphs [67, 69].

### Quality of evidence

The quality of the studies included in this review, with regards to the risk of bias, was generally assessed as moderate. It is important to note that increasing the quality of studies in this context can be challenging, as randomised controlled trials are often not feasible or ethically permissible for evaluating policy-related public health interventions, such as health insurance schemes. The absence of randomisation in the allocation of the intervention to participants can introduce various forms of bias, including confounding, which may impact the validity of the study results. Recognizing this, it is essential to thoroughly assess potential drawbacks and biases using appropriate tools [30, 31]. The authors tried to overcome this likely consequence of non-randomisation by adjusting for confounders; however, it is difficult to control for all the likely bias. The overall quality of a study can be improved through the randomisation of the intervention (where possible) and the use of causal inference statistical methods that address the potential selection problems that may arise [67, 69].

### Future research

Although we find that health insurance has a positive impact on the utilisation of ANC; we should be conscious of the intersectionality of evidence. Health insurance interventions may have varying effects across different subgroups within the population. Factors such as age, economic status, and the rural/urban setting can influence how individuals experience and benefit from health insurance coverage [47, 70]. A study by Barasa et al. reported that most insurance schemes in sub–Saharan Africa are pro-rich and have minimal benefits for the poor given the low insurance coverage [71]. The impact of health insurance schemes on utilization and financial protection may vary based on the characteristics/features of the schemes for example organization/design, implementation, enrolment levels, premiums, target population, benefit package [21, 72]. If countries are to advance UHC, there is need to understand the intersectionality of the impact, thus conduct more research to investigate the impact of health insurance across geographical domains (rural/urban), across type and level of health providers (private vs public; community-level providers vs secondary- and tertiary-level providers) and vulnerable population subgroups (e.g., people in lower socio-economic quintiles).

## Limitations

Our review included studies that were published in English after 2009, which could have led to the omission of studies published in other languages, such as those conducted in French-speaking countries in West Africa or studies before 2010 that may have reported relevant results. We acknowledge that in some contexts, individuals may have private health insurance in addition to the type of health insurance examined in this study, which may have affected the results reported in the included studies. We included studies of various designs, which may have led to variations in the interpretation of results. The use of different covariates in the models employed by the studies could have influenced the magnitude of the reported impact of health insurance.

This study focused on the direction of impact (positive, no change or negative) and significance level of the impact but did not cover the magnitude of the impact. Furthermore, due to the heterogeneity in study design and other characteristics of the included studies, it was not feasible for us to conduct a meta-analysis.

The majority of the included studies used pre-existing datasets to estimate the impact of health insurance. The datasets utilised in this regard were not developed or collected to specifically evaluate health insurance schemes. Such datasets may not be comprehensive in collecting all the relevant data points needed for a robust evaluation of the impact of health insurance.

The quality assessment of the studies was conducted using the ROBINS-I tool – a validated tool recommended by Cochrane for the quality assessment of non-randomised studies [20]. However the tool does not address problems relating to imprecision of results, where statistical analyses fail to account for clustering or matching of participants [31]. Such shortfalls may have been overlooked. Therefore, studies that were found to have serious risk of bias were not included in the causal impact analysis, to avoid increasing biases in the summary results.

## Conclusion

This review finds evidence supporting the positive impact of health insurance on the utilisation of ANC and delivery care services in low- and lower middle-income settings specially regarding receiving at least four ANC visits, delivery at a health facility and having a delivery assisted by a skilled attendant. Health insurance may contribute to making progress towards UHC, through improving access and utilisation of health services for all. The evidence on financial protection, contraception, and postnatal care is limited and inconclusive. Future evaluations of the impact of health insurance are crucial for countries to identify areas that require improvement, particularly in terms of its impact on vulnerable groups. Further research is needed to assess the impact of health insurance on contraception, postnatal care, and the financial protection of women seeking maternal and reproductive health services. Such work would contribute to a deeper understanding of the potential benefits and limitations of health insurance in these critical areas.

### Supplementary Information

**Supplementary Material 1.**

## Data Availability

The extracted data analysed during the current study is available from corresponding author on reasonable request.

## References

[1] World Health Organisation. Universal health coverage (UHC). Published April 1, 2021. https://www.who.int/news-room/fact-sheets/detail/universal-health-coverage-(uhc). Accessed 12 Jun 2022.

[2] World Health Organization. Coverage of essential health services (SDG 3.8.1). Published 2022. https://www.who.int/data/gho/data/themes/topics/service-coverage. Accessed 18 December 2022.

[3] World Health Organisation. UHC service coverage index (3.8.1). Published 2023. https://www.who.int/data/gho/indicator-metadata-registry/imr-details/4834. Accessed 20 May 2023.

[4] United Nations. SDG indicator metadata. https://unstats.un.org/sdgs/metadata/files/Metadata-03-08-01.pdf. Accessed 12 Jun 2022.

[5] World Bank, World Health Organization. Global Monitoring Report on Financial Protection in Health 2021. https://www.who.int/publications/i/item/9789240040953. Accessed 11 Jun 2023.

[6] Wagstaff A. Social Health Insurance Vs. Tax-Financed Health Systems - Evidence From The OECD. Published online 22 Jan 2013. 10.1596/1813-9450-4821.

[7] Gabani J, Mazumdar S, Suhrcke M. The effect of health financing systems on health system outcomes: a cross-country panel analysis. Health Econ. Published online 8 Dec 2022. 10.1002/HEC.4635. 10.1002/hec.4635 PMC10107855 36480236

[8] Fusheini A. Healthcare Financing Reforms: Ghana’s National Health Insurance. Heal Reforms Across World. Published online March 2020:25–54. 10.1142/9789811208928_0002.

[9] Masiye F, Chansa C (2019). Health Financing in Zambia.

[10] Cashin C, Dossou J-P. Can National Health Insurance Pave the Way to Universal Health Coverage in Sub-Saharan Africa? 2021;7(1). 10.1080/23288604.2021.2006122. 10.1080/23288604.2021.2006122 34965364

[11] Gottret P, Schieber G. Health financing revisited: a practitioner’s guide. 2006. https://documents.worldbank.org/en/publication/documents-reports/documentdetail/874011468313782370/health-financing-revisited-a-practitioners-guide. Accessed 18 May 2023.

[12] World Health Assembly 58. Social Health Insurance: Sustainable Health Financing, Universal Coverage and Social Health Insurance: Report by the Secretariat. 2005. https://apps.who.int/iris/handle/10665/20302. Accessed 5 June 2023.

[13] Government of India. Rashtriya Swasthya Bima Yojana. https://www.india.gov.in/spotlight/rashtriya-swasthya-bima-yojana. Accessed 20 May 2023.

[14] Ranjan A, Dixit P, Mukhopadhyay I, Thiagarajan S (2018). Effectiveness of government strategies for financial protection against costs of hospitalization Care in India. BMC Public Health.

[15] United Nations (UN). United Nations Millennium Development Goals. https://www.un.org/millenniumgoals/. Accessed 5 Jun 2023.

[16] Watkins DA, Jamison DT, Mills A, et al. Universal Health Coverage and Essential Packages of Care. In: Disease Control Priorities, Third Edition (Volume 9): Improving Health and Reducing Poverty. The World Bank. 2017:43–65. 10.1596/978-1-4648-0527-1_ch3. 30212154

[17] Kaiser AH, Ekman B, Dimarco M, Sundewall J (2021). The cost-effectiveness of sexual and reproductive health and rights interventions in low- and middle-income countries: a scoping review. Sex Reprod Heal Matters.

[18] World Health Organisation. Maternal mortality fact sheet. Published September 2019. https://www.who.int/news-room/fact-sheets/detail/maternal-mortality. Accessed 9 June 2022.

[19] UN Women. Universal Health Coverage, Gender Equality and Social Protection. A Health Systems Approach. 2020. https://www.unwomen.org/sites/default/files/Headquarters/Attachments/Sections/Library/Publications/2020/Discussion-paper-Universal-health-coverage-gender-equality-and-social-protection-en.pdf. Accessed 22 May 2023.

[20] Spaan E, Mathijssen J, Tromp N, McBain F, ten Have A, Baltussen R (2012). The impact of health insurance in Africa and Asia: a systematic review. Bull World Health Organ.

[21] Erlangga D, Suhrcke M, Ali S, Bloor K (2019). The impact of public health insurance on health care utilisation, financial protection and health status in low- And middle-income countries: A systematic review. PLoS ONE.

[22] Acharya A, Vellakkal S, Taylor F (2013). The impact of health insurance schemes for the informal sector in low-and middle-income countries: A systematic review. World Bank Res Obs.

[23] Zhang C, Fu C, Song Y, Feng R, Wu X, Li Y (2020). Utilization of public health care by people with private health insurance: A systematic review and meta-analysis. BMC Public Health.

[24] Comfort AB, Peterson LA, Hatt LE (2013). Effect of Health Insurance on the Use and Provision of Maternal Health Services and Maternal and Neonatal Health Outcomes: A Systematic Review. J Health Popul Nutr.

[25] Page MJ, McKenzie JE, Bossuyt PM, The PRISMA (2020). statement: an updated guideline for reporting systematic reviews. BMJ.

[26] Schardt C, Adams MB, Owens T, Keitz S, Fontelo P. Utilization of the PICO framework to improve searching PubMed for clinical questions. BMC Med Inform Decis Mak. 2007;7. 10.1186/1472-6947-7-16. 10.1186/1472-6947-7-16 PMC1904193 17573961

[27] World Bank Group. World Bank Country and Lending Groups – World Bank Data. Published 2021. https://datahelpdesk.worldbank.org/knowledgebase/articles/906519. Accessed 11 Aug 2021.

[28] Moher D, Liberati A, Tetzlaff J (2009). Preferred reporting items for systematic reviews and meta-analyses: The PRISMA statement (Chinese edition). J Chinese Integr Med.

[29] Veritas Health Innovation. Covidence systematic review software. https://www.covidence.org/. Accessed 31 Dec 2021.

[30] Sterne JA, Hernán MA, McAleenan A, Reeves BC. Chapter 25: assessing risk of bias in a non-randomized study | Cochrane Training. Cochrane. https://training.cochrane.org/handbook/current/chapter-25. Accessed 20 Dec 2022.

[31] Sterne JA, Hernán MA, Reeves BC, et al. ROBINS-I: a tool for assessing risk of bias in non-randomised studies of interventions. BMJ. 2016;355. 10.1136/BMJ.I4919. 10.1136/bmj.i4919 PMC5062054 27733354

[32] Ma LL, Wang YY, Yang ZH, Huang D, Weng H, Zeng XT (2020). Methodological quality (risk of bias) assessment tools for primary and secondary medical studies: What are they and which is better?. Mil Med Res.

[33] Schünemann HJ, Cuello C, Akl EA (2019). GRADE guidelines: 18. How ROBINS-I and other tools to assess risk of bias in nonrandomized studies should be used to rate the certainty of a body of evidence. J Clin Epidemiol..

[34] Samarakoon S, Parinduri RA. The effects of social health insurance on women’s healthcare use: evidence from Indonesia. Singapore Econ Rev . Published online Dec 18, 2020. 10.1142/S0217590820500733.

[35] Agbanyo R, Peprah JA (2021). National health insurance and the choice of delivery facility among expectant mothers in Ghana. Int J Heal Econ Manag.

[36] Ravit M, Ravalihasy A, Audibert M (2020). The impact of the obstetrical risk insurance scheme in Mauritania on maternal healthcare utilization: a propensity score matching analysis. Health Policy Plan.

[37] Chang AY, Li Y, Ogbuoji O (2018). Modest improvements in skilled birth attendants at delivery with increased Mutuelles coverage in Rwanda. J Public Health (Bangkok).

[38] Rashad AS, Sharaf MF, Mansour EI (2019). Does Public Health Insurance Increase Maternal Health Care Utilization in Egypt?. J Int Dev.

[39] Gouda HN, Hodge A, Bermejo R, Zeck W, Jimenez-Soto E (2016). The Impact of Healthcare Insurance on the Utilisation of Facility-Based Delivery for Childbirth in the Philippines. PLoS ONE.

[40] Philibert A, Ravit M, Ridde V (2017). Maternal and neonatal health impact of obstetrical risk insurance scheme in Mauritania: a quasi experimental before-and-after study. Health Policy Plan.

[41] Anindya K, Lee JT, McPake B, Wilopo SA, Millett C, Carvalho N. Impact of Indonesia’s national health insurance scheme on inequality in access to maternal health services: a propensity score matched analysis. J Glob Health. 2020;10(1). 10.7189/JOGH.10.010429. 10.7189/jogh.10.010429 PMC7298736 32566167

[42] Kuwawenaruwa A, Ramsey K, Binyaruka P, Baraka J, Manzi F, Borghi J (2019). Implementation and effectiveness of free health insurance for the poor pregnant women in Tanzania: A mixed methods evaluation. Soc Sci Med.

[43] Aizawa T (2019). The impact of health insurance on out-of-pocket expenditure on delivery in Indonesia..

[44] Bonfrer I, Breebaart L, De Poel E, Van. (2016). The Effects of Ghana’s National Health Insurance Scheme on Maternal and Infant Health Care Utilization. PLoS ONE.

[45] El Omari S, Karasneh M (2021). Social health insurance in the Philippines: do the poor really benefit?. J Econ Financ.

[46] Wang W, Temsah G, Mallick L (2017). The impact of health insurance on maternal health care utilization: evidence from Ghana. Indonesia and Rwanda Health Policy Plan.

[47] Kofinti RE, Asmah EE, Ameyaw EK (2022). Comparative study of the effect of National Health Insurance Scheme on use of delivery and antenatal care services between rural and urban women in Ghana. Health Econ Rev.

[48] Bousmah MAQ, Diakhaté P, Toulao GÀD, Le Hesran JY, Lalou R (2022). Effects of a free health insurance programme for the poor on health service utilisation and financial protection in Senegal. BMJ Glob Heal.

[49] Mussa EC, Palermo T, Angeles G (2023). Impact of community-based health insurance on health services utilisation among vulnerable households in Amhara region. Ethiopia BMC Health Serv Res.

[50] Garg S, Tripathi N, Bebarta KK (2023). Does government health insurance protect households from out of pocket expenditure and distress financing for caesarean and non-caesarean institutional deliveries in India? Findings from the national family health survey (2019–21). BMC Res Notes.

[51] Harris AD, McGregor JC, Perencevich EN (2006). The Use and Interpretation of Quasi-Experimental Studies in Medical Informatics. J Am Med Inform Assoc.

[52] The Demographic and Health Surveys (DHS) Program. The Demographic and Health Surveys (DHS) Program. https://dhsprogram.com/. Accessed 3, Jul 2022.

[53] Bauserman M, Thorsten VR, Nolen TL (2020). Maternal mortality in six low and lower-middle income countries from 2010 to 2018: risk factors and trends. Reprod Health.

[54] Goldenberg RL, McClure EM, Saleem S. Improving pregnancy outcomes in low- and middle-income countries. Reprod Health. 2018;15(Suppl 1). 10.1186/S12978-018-0524-5. 10.1186/s12978-018-0524-5 PMC6019988 29945628

[55] Horton S, Levin C. Cost-Effectiveness of Interventions for Reproductive, Maternal, Neonatal, and Child Health. In: Disease Control Priorities, Third Edition (Volume 2): Reproductive, Maternal, Newborn, and Child Health. The World Bank. 2016:319–334. 10.1596/978-1-4648-0348-2_ch17. 27227227

[56] The World BanK. Out-of-pocket expenditure (% of current health expenditure). https://data.worldbank.org/indicator/SH.XPD.OOPC.CH.ZS. Accessed 19 Jun 2022.

[57] Vu PH, Sepehri A, Tran LTT (2022). Trends in out-of-pocket expenditure on facility-based delivery and financial protection of health insurance: findings from Vietnam’s Household Living Standard Survey 2006–2018. Int J Heal Econ Manag.

[58] International Planned Parenthood Federation. Ghana offers free long-term contraception in a ‘game changer’ for women’s reproductive health rights. Published 2022. https://www.ippf.org/blogs/ghana-offers-free-long-term-contraception-game-changer-womens-reproductive-health-rights. Accessed 6 June 2023.

[59] Priyadarshani P, Borovac-Pinheiro A, Burke TF. Glob J Reprod Med Access and Quality of Postnatal Care in Low-and Middle-Income Countries: a Pragmatic Review. J Reprod Med. 2021;8(3). 10.19080/GJORM.2021.08.5556740.

[60] Sacks E, Finlayson K, Brizuela V (2022). Factors that influence uptake of routine postnatal care: Findings on women’s perspectives from a qualitative evidence synthesis. PLoS ONE.

[61] Clarke-Deelder E, Opondo K, Oguttu M, Burke T, Cohen JL, McConnell M (2023). Immediate postpartum care in low- and middle-income countries: A gap in healthcare quality research and practice. Am J Obstet Gynecol MFM.

[62] Beeckman K, Louckx F, Downe S, Putman K (2013). The relationship between antenatal care and preterm birth: the importance of content of care. Eur J Public Health.

[63] Pena-Rosas JP, Lawrie T, Tunçalp (2017). WHO recommendations on antenatal care for a positive pregnancy experience-going beyond survival. BJOG..

[64] World Health Health Organisation. WHO Recommendations on antenatal care for a positive pregnancy experience. 2016. https://www.who.int/publications/i/item/9789241549912. Accessed 28 May 2023. 28079998

[65] World Health Organisation. WHO recommendations on maternal and newborn care for a positive postnatal experience: executive summary. Published 2022. https://www.who.int/publications/i/item/9789240044074. Accessed 19 Jun 2022. 35467813

[66] Wojcieszek AM, Bonet M, Portela A (2023). WHO recommendations on maternal and newborn care for a positive postnatal experience: strengthening the maternal and newborn care continuum. BMJ Glob Heal.

[67] Abadie A, Cattaneo MD (2018). Econometric Methods for Program Evaluation. Published online.

[68] Abadie A, Imbens GW (2012). Bias-Corrected Matching Estimators for Average Treatment Effects. J Bus Econ Stat.

[69] Cunningham S (2021). Causal Inference The Mixtape - 3 Directed Acyclic Graphs. Causal Inference.

[70] Ronsmans C, Graham WJ (2006). Maternal mortality: who, when, where, and why. Lancet.

[71] Barasa E, Kazungu J, Nguhiu P, Ravishankar N (2021). Examining the level and inequality in health insurance coverage in 36 sub-Saharan African countries. BMJ Glob Heal.

[72] Eze P, Ilechukwu S, Lawani LO (2023). Impact of community-based health insurance in low- and middle-income countries: a systematic review and meta-analysis. Vojnov L, ed. PLoS One..

