# Supplementary material for: The impact of health insurance on maternal and reproductive health service utilization and financial protection in low- and lower middle-income countries: a systematic review of the evidence
Source: BMC Health Serv Res. 2024 Apr 5;24:432. doi: 10.1186/s12913-024-10815-5 (PMC10996233; doi:10.1186/s12913-024-10815-5)
Supplement: Supplementary file 1 — Supplementary Material 1. [file 12913_2024_10815_MOESM1_ESM.docx]

**APPENDIX**

**Appendix 1: Search Strategy customised to specific databases**

**PubMed search string**

("developing countries"[MeSH Terms] OR ("developing"[All Fields] AND "countries"[All Fields]) OR "developing countries"[All Fields] OR ("low"[All Fields] AND "income"[All Fields] AND "country"[All Fields]) OR "low income country"[All Fields] OR ("developing countries"[MeSH Terms] OR ("developing"[All Fields] AND "countries"[All Fields]) OR "developing countries"[All Fields] OR ("lower"[All Fields] AND "middle"[All Fields] AND "income"[All Fields] AND "country"[All Fields]) OR "lower middle income country"[All Fields]) OR ("low"[All Fields] AND ("developing countries"[MeSH Terms] OR ("developing"[All Fields] AND "countries"[All Fields]) OR "developing countries"[All Fields] OR ("lower"[All Fields] AND "middle"[All Fields] AND "income"[All Fields] AND "countries"[All Fields]) OR "lower middle income countries"[All Fields])) OR ("developing countries"[MeSH Terms] OR ("developing"[All Fields] AND "countries"[All Fields]) OR "developing countries"[All Fields] OR ("developing"[All Fields] AND "country"[All Fields]) OR "developing country"[All Fields]) OR ("low"[All Fields] AND ("health resources"[MeSH Terms] OR ("health"[All Fields] AND "resources"[All Fields]) OR "health resources"[All Fields] OR "resource"[All Fields] OR "resources"[All Fields] OR "resource s"[All Fields] OR "resourced"[All Fields] OR "resourceful"[All Fields] OR "resourcefulness"[All Fields] OR "resourcing"[All Fields]) AND ("setting"[All Fields] OR "setting s"[All Fields] OR "settings"[All Fields])) OR ("developing countries"[MeSH Terms] OR ("developing"[All Fields] AND "countries"[All Fields]) OR "developing countries"[All Fields] OR ("developing"[All Fields] AND "nations"[All Fields]) OR "developing nations"[All Fields]) OR ("developing countries"[MeSH Terms] OR ("developing"[All Fields] AND "countries"[All Fields]) OR "developing countries"[All Fields] OR ("less"[All Fields] AND "developed"[All Fields] AND "country"[All Fields]) OR "less developed country"[All Fields]) OR ("developing countries"[MeSH Terms] OR ("developing"[All Fields] AND "countries"[All Fields]) OR "developing countries"[All Fields] OR ("under"[All Fields] AND "developed"[All Fields] AND "country"[All Fields]) OR "under developed country"[All Fields]) OR ("afghanistan"[MeSH Terms] OR "afghanistan"[All Fields] OR "afghanistan s"[All Fields]) OR ("guinea bissau"[MeSH Terms] OR "guinea bissau"[All Fields] OR ("guinea"[All Fields] AND "bissau"[All Fields]) OR "guinea bissau"[All Fields]) OR ("somalia"[MeSH Terms] OR "somalia"[All Fields] OR "somalia s"[All Fields]) OR ("burkina faso"[MeSH Terms] OR ("burkina"[All Fields] AND "faso"[All Fields]) OR "burkina faso"[All Fields]) OR ("democratic people s republic of korea"[MeSH Terms] OR ("democratic"[All Fields] AND "people s"[All Fields] AND "republic"[All Fields] AND "korea"[All Fields]) OR "democratic people s republic of korea"[All Fields] OR ("north"[All Fields] AND "korea"[All Fields]) OR "north korea"[All Fields]) OR (("people s"[All Fields] OR "peopled"[All Fields] OR "peopling"[All Fields] OR "persons"[MeSH Terms] OR "persons"[All Fields] OR "people"[All Fields] OR "peoples"[All Fields]) AND ("democrat"[All Fields] OR "democratic"[All Fields] OR "democratically"[All Fields] OR "democratization"[All Fields] OR "democratize"[All Fields] OR "democratized"[All Fields] OR "democratizing"[All Fields] OR "democrats"[All Fields]) AND ("republic of korea"[MeSH Terms] OR ("republic"[All Fields] AND "korea"[All Fields]) OR "republic of korea"[All Fields])) OR ("south sudan"[MeSH Terms] OR ("south"[All Fields] AND "sudan"[All Fields]) OR "south sudan"[All Fields]) OR ("burundi"[MeSH Terms] OR "burundi"[All Fields]) OR ("liberia"[MeSH Terms] OR "liberia"[All Fields] OR "liberia s"[All Fields]) OR ("sudan"[MeSH Terms] OR "sudan"[All Fields] OR "sudans"[All Fields] OR "sudan s"[All Fields]) OR ("central african republic"[MeSH Terms] OR ("central"[All Fields] AND "african"[All Fields] AND "republic"[All Fields]) OR "central african republic"[All Fields]) OR ("madagascar"[MeSH Terms] OR "madagascar"[All Fields] OR "madagascar s"[All Fields]) OR ("syria"[MeSH Terms] OR "syria"[All Fields] OR "syria s"[All Fields]) OR ("chad"[MeSH Terms] OR "chad"[All Fields]) OR ("malawi"[MeSH Terms] OR "malawi"[All Fields] OR "malawi s"[All Fields]) OR ("togo"[MeSH Terms] OR "togo"[All Fields]) OR (("democrat"[All Fields] OR "democratic"[All Fields] OR "democratically"[All Fields] OR "democratization"[All Fields] OR "democratize"[All Fields] OR "democratized"[All Fields] OR "democratizing"[All Fields] OR "democrats"[All Fields]) AND ("republic"[All Fields] OR "republic s"[All Fields] OR "republics"[All Fields]) AND ("congo"[MeSH Terms] OR "congo"[All Fields])) OR "DRC"[All Fields] OR ("mali"[MeSH Terms] OR "mali"[All Fields]) OR ("uganda"[MeSH Terms] OR "uganda"[All Fields] OR "uganda s"[All Fields]) OR "Eriteria"[All Fields] OR ("mozambique"[MeSH Terms] OR "mozambique"[All Fields] OR "mozambique s"[All Fields]) OR ("yemen"[MeSH Terms] OR "yemen"[All Fields]) OR ("ethiopia"[MeSH Terms] OR "ethiopia"[All Fields] OR "ethiopia s"[All Fields]) OR ("niger"[MeSH Terms] OR "niger"[All Fields]) OR ("gambia"[MeSH Terms] OR "gambia"[All Fields] OR "gambia s"[All Fields]) OR ("rwanda"[MeSH Terms] OR "rwanda"[All Fields] OR "rwanda s"[All Fields]) OR ("guinea"[MeSH Terms] OR "guinea"[All Fields] OR "guinea s"[All Fields] OR "guineas"[All Fields]) OR ("sierra leone"[MeSH Terms] OR ("sierra"[All Fields] AND "leone"[All Fields]) OR "sierra leone"[All Fields]) OR ("angola"[MeSH Terms] OR "angola"[All Fields] OR "angola s"[All Fields]) OR ("honduras"[MeSH Terms] OR "honduras"[All Fields]) OR ("philippine"[All Fields] OR "philippines"[MeSH Terms] OR "philippines"[All Fields]) OR ("algeria"[MeSH Terms] OR "algeria"[All Fields]) OR ("india"[MeSH Terms] OR "india"[All Fields] OR "india s"[All Fields] OR "indias"[All Fields]) OR ("samoa"[MeSH Terms] OR "samoa"[All Fields] OR "samoas"[All Fields]) OR ("bangladesh"[MeSH Terms] OR "bangladesh"[All Fields] OR "bangladesh s"[All Fields]) OR ("indonesia"[MeSH Terms] OR "indonesia"[All Fields] OR "indonesia s"[All Fields] OR "indonesias"[All Fields]) OR ("Sao"[All Fields] AND "Tome"[All Fields]) OR ("principe"[All Fields] OR "principes"[All Fields]) OR ("belize"[MeSH Terms] OR "belize"[All Fields]) OR ("iran"[MeSH Terms] OR "iran"[All Fields]) OR ("senegal"[MeSH Terms] OR "senegal"[All Fields] OR "senegal s"[All Fields]) OR ("benin"[MeSH Terms] OR "benin"[All Fields] OR "benin s"[All Fields]) OR ("kenya"[MeSH Terms] OR "kenya"[All Fields] OR "kenya s"[All Fields]) OR ("melanesia"[MeSH Terms] OR "melanesia"[All Fields] OR ("solomon"[All Fields] AND "islands"[All Fields]) OR "solomon islands"[All Fields]) OR ("bhutan"[MeSH Terms] OR "bhutan"[All Fields] OR "bhutan s"[All Fields]) OR ("micronesia"[MeSH Terms] OR "micronesia"[All Fields] OR "kiribati"[All Fields]) OR ("sri lanka"[MeSH Terms] OR ("sri"[All Fields] AND "lanka"[All Fields]) OR "sri lanka"[All Fields]) OR ("bolivia"[MeSH Terms] OR "bolivia"[All Fields]) OR ("kyrgyz"[All Fields] OR "kyrgyzes"[All Fields]) OR ("tanzania"[MeSH Terms] OR "tanzania"[All Fields] OR "tanzania s"[All Fields]) OR ("cabo verde"[MeSH Terms] OR ("cabo"[All Fields] AND "verde"[All Fields]) OR "cabo verde"[All Fields]) OR ("Lao"[All Fields] AND "PDR"[All Fields]) OR ("laos"[MeSH Terms] OR "laos"[All Fields]) OR ("tajikistan"[MeSH Terms] OR "tajikistan"[All Fields]) OR ("cambodia"[MeSH Terms] OR "cambodia"[All Fields] OR "cambodia s"[All Fields]) OR ("lesotho"[MeSH Terms] OR "lesotho"[All Fields]) OR ("timor leste"[MeSH Terms] OR "timor leste"[All Fields] OR ("timor"[All Fields] AND "leste"[All Fields]) OR "timor leste"[All Fields]) OR ("cameroon"[MeSH Terms] OR "cameroon"[All Fields] OR "cameroons"[All Fields] OR "cameroon s"[All Fields]) OR ("mauritania"[MeSH Terms] OR "mauritania"[All Fields]) OR ("tunisia"[MeSH Terms] OR "tunisia"[All Fields]) OR ("comoros"[MeSH Terms] OR "comoros"[All Fields] OR "comoro"[All Fields]) OR ("micronesia"[MeSH Terms] OR "micronesia"[All Fields]) OR ("ukraine"[MeSH Terms] OR "ukraine"[All Fields] OR "ukraine s"[All Fields]) OR ("congo"[MeSH Terms] OR "congo"[All Fields]) OR ("mongolia"[MeSH Terms] OR "mongolia"[All Fields] OR "mongolia s"[All Fields]) OR ("uzbekistan"[MeSH Terms] OR "uzbekistan"[All Fields]) OR ("cote d ivoire"[MeSH Terms] OR ("cote"[All Fields] AND "d ivoire"[All Fields]) OR "cote d ivoire"[All Fields] OR ("ivory"[All Fields] AND "coast"[All Fields]) OR "ivory coast"[All Fields]) OR ("morocco"[MeSH Terms] OR "morocco"[All Fields]) OR ("vanuatu"[MeSH Terms] OR "vanuatu"[All Fields]) OR ("djibouti"[MeSH Terms] OR "djibouti"[All Fields]) OR ("myanmar"[MeSH Terms] OR "myanmar"[All Fields] OR "myanmar s"[All Fields] OR "myanmars"[All Fields]) OR ("vietnam"[MeSH Terms] OR "vietnam"[All Fields] OR "vietnam s"[All Fields]) OR ("egypt"[MeSH Terms] OR "egypt"[All Fields] OR "egypt s"[All Fields]) OR ("nepal"[MeSH Terms] OR "nepal"[All Fields] OR "nepal s"[All Fields]) OR ("middle east"[MeSH Terms] OR ("middle"[All Fields] AND "east"[All Fields]) OR "middle east"[All Fields] OR ("west"[All Fields] AND "bank"[All Fields]) OR "west bank"[All Fields]) OR "Gaza"[All Fields] OR ("el salvador"[MeSH Terms] OR ("el"[All Fields] AND "salvador"[All Fields]) OR "el salvador"[All Fields]) OR ("nicaragua"[MeSH Terms] OR "nicaragua"[All Fields] OR "nicaragua s"[All Fields]) OR ("zambia"[MeSH Terms] OR "zambia"[All Fields] OR "zambia s"[All Fields]) OR ("eswatini"[MeSH Terms] OR "eswatini"[All Fields]) OR ("nigeria"[MeSH Terms] OR "nigeria"[All Fields] OR "nigeria s"[All Fields]) OR ("zimbabwe"[MeSH Terms] OR "zimbabwe"[All Fields] OR "zimbabwe s"[All Fields]) OR ("ghana"[MeSH Terms] OR "ghana"[All Fields] OR "ghana s"[All Fields]) OR ("pakistan"[MeSH Terms] OR "pakistan"[All Fields] OR "pakistan s"[All Fields]) OR ("haiti"[MeSH Terms] OR "haiti"[All Fields] OR "haiti s"[All Fields]) OR ("papua new guinea"[MeSH Terms] OR ("papua"[All Fields] AND "new"[All Fields] AND "guinea"[All Fields]) OR "papua new guinea"[All Fields])) AND ("impact"[All Fields] OR "impactful"[All Fields] OR "impacting"[All Fields] OR "impacts"[All Fields] OR "tooth, impacted"[MeSH Terms] OR ("tooth"[All Fields] AND "impacted"[All Fields]) OR "impacted tooth"[All Fields] OR "impacted"[All Fields] OR ("effect"[All Fields] OR "effecting"[All Fields] OR "effective"[All Fields] OR "effectively"[All Fields] OR "effectiveness"[All Fields] OR "effectivenesses"[All Fields] OR "effectives"[All Fields] OR "effectivities"[All Fields] OR "effectivity"[All Fields] OR "effects"[All Fields]) OR (("therapeutics"[MeSH Terms] OR "therapeutics"[All Fields] OR "treatments"[All Fields] OR "therapy"[MeSH Subheading] OR "therapy"[All Fields] OR "treatment"[All Fields] OR "treatment s"[All Fields]) AND ("effect"[All Fields] OR "effecting"[All Fields] OR "effective"[All Fields] OR "effectively"[All Fields] OR "effectiveness"[All Fields] OR "effectivenesses"[All Fields] OR "effectives"[All Fields] OR "effectivities"[All Fields] OR "effectivity"[All Fields] OR "effects"[All Fields])) OR (("causal"[All Fields] OR "causality"[MeSH Terms] OR "causality"[All Fields] OR "causalities"[All Fields] OR "causally"[All Fields] OR "etiology"[MeSH Subheading] OR "etiology"[All Fields]) AND ("impact"[All Fields] OR "impactful"[All Fields] OR "impacting"[All Fields] OR "impacts"[All Fields] OR "tooth, impacted"[MeSH Terms] OR ("tooth"[All Fields] AND "impacted"[All Fields]) OR "impacted tooth"[All Fields] OR "impacted"[All Fields])) OR ("outcome"[All Fields] OR "outcomes"[All Fields]) OR ("influence"[All Fields] OR "influenced"[All Fields] OR "influences"[All Fields] OR "influencing"[All Fields]) OR ("associate"[All Fields] OR "associated"[All Fields] OR "associates"[All Fields] OR "associating"[All Fields] OR "association"[MeSH Terms] OR "association"[All Fields] OR "associations"[All Fields]) OR ("affect"[MeSH Terms] OR "affect"[All Fields] OR "affects"[All Fields] OR "affected"[All Fields] OR "affecteds"[All Fields] OR "affecting"[All Fields]) OR ("relationship"[All Fields] OR "relationships"[All Fields]) OR ("infer"[All Fields] OR "inferability"[All Fields] OR "inferable"[All Fields] OR "inference"[All Fields] OR "inferences"[All Fields] OR "inferred"[All Fields] OR "inferring"[All Fields] OR "infers"[All Fields])) AND ("insurability"[All Fields] OR "insurable"[All Fields] OR "insurance carriers"[MeSH Terms] OR ("insurance"[All Fields] AND "carriers"[All Fields]) OR "insurance carriers"[All Fields] OR "insurer"[All Fields] OR "insurers"[All Fields] OR "insurance"[MeSH Terms] OR "insurance"[All Fields] OR "insurances"[All Fields] OR "insurance s"[All Fields] OR "insurant"[All Fields] OR "insurants"[All Fields] OR "insure"[All Fields] OR "insured"[All Fields] OR "insured s"[All Fields] OR "insureds"[All Fields] OR "insurer s"[All Fields] OR "insures"[All Fields] OR "insuring"[All Fields] OR (("governability"[All Fields] OR "governable"[All Fields] OR "governance"[All Fields] OR "governances"[All Fields] OR "government"[MeSH Terms] OR "government"[All Fields] OR "governments"[All Fields] OR "government s"[All Fields]) AND ("economics"[MeSH Subheading] OR "economics"[All Fields] OR "funding"[All Fields] OR "economics"[MeSH Terms] OR "financial management"[MeSH Terms] OR ("financial"[All Fields] AND "management"[All Fields]) OR "financial management"[All Fields] OR "funded"[All Fields] OR "funds"[All Fields] OR "fund s"[All Fields] OR "fundings"[All Fields]) AND ("insurance, health"[MeSH Terms] OR ("insurance"[All Fields] AND "health"[All Fields]) OR "health insurance"[All Fields] OR ("health"[All Fields] AND "insurance"[All Fields]))) OR ("insurance, health"[MeSH Terms] OR ("insurance"[All Fields] AND "health"[All Fields]) OR "health insurance"[All Fields] OR ("health"[All Fields] AND "insurance"[All Fields])) OR (("public health"[MeSH Terms] OR ("public"[All Fields] AND "health"[All Fields]) OR "public health"[All Fields]) AND ("insurability"[All Fields] OR "insurable"[All Fields] OR "insurance carriers"[MeSH Terms] OR ("insurance"[All Fields] AND "carriers"[All Fields]) OR "insurance carriers"[All Fields] OR "insurer"[All Fields] OR "insurers"[All Fields] OR "insurance"[MeSH Terms] OR "insurance"[All Fields] OR "insurances"[All Fields] OR "insurance s"[All Fields] OR "insurant"[All Fields] OR "insurants"[All Fields] OR "insure"[All Fields] OR "insured"[All Fields] OR "insured s"[All Fields] OR "insureds"[All Fields] OR "insurer s"[All Fields] OR "insures"[All Fields] OR "insuring"[All Fields])) OR (("social behavior"[MeSH Terms] OR ("social"[All Fields] AND "behavior"[All Fields]) OR "social behavior"[All Fields] OR "sociality"[All Fields] OR "social"[All Fields] OR "socialisation"[All Fields] OR "socialization"[MeSH Terms] OR "socialization"[All Fields] OR "socialise"[All Fields] OR "socialised"[All Fields] OR "socialising"[All Fields] OR "socialities"[All Fields] OR "socializations"[All Fields] OR "socialize"[All Fields] OR "socialized"[All Fields] OR "socializers"[All Fields] OR "socializes"[All Fields] OR "socializing"[All Fields] OR "socially"[All Fields] OR "socials"[All Fields]) AND ("insurance, health"[MeSH Terms] OR ("insurance"[All Fields] AND "health"[All Fields]) OR "health insurance"[All Fields] OR ("health"[All Fields] AND "insurance"[All Fields]))) OR ("national health programs"[MeSH Terms] OR ("national"[All Fields] AND "health"[All Fields] AND "programs"[All Fields]) OR "national health programs"[All Fields] OR ("national"[All Fields] AND "health"[All Fields] AND "insurance"[All Fields]) OR "national health insurance"[All Fields]) OR ("community based health insurance"[MeSH Terms] OR ("community based"[All Fields] AND "health"[All Fields] AND "insurance"[All Fields]) OR "community based health insurance"[All Fields] OR ("community"[All Fields] AND "health"[All Fields] AND "insurance"[All Fields]) OR "community health insurance"[All Fields]) OR (("geographic locations"[MeSH Terms] OR ("geographic"[All Fields] AND "locations"[All Fields]) OR "geographic locations"[All Fields] OR "region"[All Fields] OR "region s"[All Fields] OR "regional"[All Fields] OR "regionalization"[All Fields] OR "regionalizations"[All Fields] OR "regionalize"[All Fields] OR "regionalized"[All Fields] OR "regionalizing"[All Fields] OR "regionally"[All Fields] OR "regionals"[All Fields] OR "regions"[All Fields]) AND ("insurance, health"[MeSH Terms] OR ("insurance"[All Fields] AND "health"[All Fields]) OR "health insurance"[All Fields] OR ("health"[All Fields] AND "insurance"[All Fields]))) OR (("risk"[MeSH Terms] OR "risk"[All Fields]) AND ("pooled"[All Fields] OR "pooling"[All Fields] OR "poolings"[All Fields]))) AND ("statistics and numerical data"[MeSH Subheading] OR ("statistics"[All Fields] AND "numerical"[All Fields] AND "data"[All Fields]) OR "statistics and numerical data"[All Fields] OR "utilization"[All Fields] OR "utilisation"[All Fields] OR "utilisations"[All Fields] OR "utilise"[All Fields] OR "utilised"[All Fields] OR "utilises"[All Fields] OR "utilising"[All Fields] OR "utilities"[All Fields] OR "utility"[All Fields] OR "utilizations"[All Fields] OR "utilize"[All Fields] OR "utilized"[All Fields] OR "utilizer"[All Fields] OR "utilizers"[All Fields] OR "utilizes"[All Fields] OR "utilizing"[All Fields] OR ("health services accessibility"[MeSH Terms] OR ("health"[All Fields] AND "services"[All Fields] AND "accessibility"[All Fields]) OR "health services accessibility"[All Fields] OR ("access"[All Fields] AND "health"[All Fields] AND "services"[All Fields]) OR "access to health services"[All Fields]) OR ("access"[All Fields] OR "accessed"[All Fields] OR "accesses"[All Fields] OR "accessibilities"[All Fields] OR "accessibility"[All Fields] OR "accessible"[All Fields] OR "accessing"[All Fields]) OR ("statistics and numerical data"[MeSH Subheading] OR ("statistics"[All Fields] AND "numerical"[All Fields] AND "data"[All Fields]) OR "statistics and numerical data"[All Fields] OR "use"[All Fields]) OR ("benefit"[All Fields] OR "benefited"[All Fields] OR "benefiting"[All Fields] OR "benefits"[All Fields] OR "benefitted"[All Fields] OR "benefitting"[All Fields]) OR ("patient acceptance of health care"[MeSH Terms] OR ("patient"[All Fields] AND "acceptance"[All Fields] AND "health"[All Fields] AND "care"[All Fields]) OR "patient acceptance of health care"[All Fields] OR ("health"[All Fields] AND "services"[All Fields] AND "utilisation"[All Fields]) OR "health services utilisation"[All Fields]) OR ("delivery of health care"[MeSH Terms] OR ("delivery"[All Fields] AND "health"[All Fields] AND "care"[All Fields]) OR "delivery of health care"[All Fields] OR "healthcare"[All Fields] OR "healthcare s"[All Fields] OR "healthcares"[All Fields]) OR ("health services"[MeSH Terms] OR ("health"[All Fields] AND "services"[All Fields]) OR "health services"[All Fields] OR ("health"[All Fields] AND "service"[All Fields]) OR "health service"[All Fields]) OR (("health"[MeSH Terms] OR "health"[All Fields] OR "health s"[All Fields] OR "healthful"[All Fields] OR "healthfulness"[All Fields] OR "healths"[All Fields]) AND "eduction"[All Fields]) OR (("uptake"[All Fields] OR "uptakes"[All Fields] OR "uptaking"[All Fields]) AND ("health services"[MeSH Terms] OR ("health"[All Fields] AND "services"[All Fields]) OR "health services"[All Fields])) OR (("health facilities"[MeSH Terms] OR ("health"[All Fields] AND "facilities"[All Fields]) OR "health facilities"[All Fields] OR ("health"[All Fields] AND "facility"[All Fields]) OR "health facility"[All Fields]) AND ("visit"[All Fields] OR "visitation"[All Fields] OR "visitations"[All Fields] OR "visited"[All Fields] OR "visiting"[All Fields] OR "visits"[All Fields])) OR (("hospital s"[All Fields] OR "hospitalisation"[All Fields] OR "hospitalization"[MeSH Terms] OR "hospitalization"[All Fields] OR "hospitalised"[All Fields] OR "hospitalising"[All Fields] OR "hospitality"[All Fields] OR "hospitalisations"[All Fields] OR "hospitalizations"[All Fields] OR "hospitalize"[All Fields] OR "hospitalized"[All Fields] OR "hospitalizing"[All Fields] OR "hospitals"[MeSH Terms] OR "hospitals"[All Fields] OR "hospital"[All Fields]) AND ("visit"[All Fields] OR "visitation"[All Fields] OR "visitations"[All Fields] OR "visited"[All Fields] OR "visiting"[All Fields] OR "visits"[All Fields])) OR ("patient acceptance of health care"[MeSH Terms] OR ("patient"[All Fields] AND "acceptance"[All Fields] AND "health"[All Fields] AND "care"[All Fields]) OR "patient acceptance of health care"[All Fields]) OR (("economics"[MeSH Terms] OR "economics"[All Fields] OR "financial"[All Fields] OR "financially"[All Fields] OR "financials"[All Fields] OR "financier"[All Fields] OR "financiers"[All Fields]) AND ("protect"[All Fields] OR "protected"[All Fields] OR "protecting"[All Fields] OR "protection"[All Fields] OR "protections"[All Fields] OR "protective agents"[Pharmacological Action] OR "protective agents"[MeSH Terms] OR ("protective"[All Fields] AND "agents"[All Fields]) OR "protective agents"[All Fields] OR "protectant"[All Fields] OR "protectants"[All Fields] OR "protective"[All Fields] OR "protectively"[All Fields] OR "protectiveness"[All Fields] OR "protectives"[All Fields] OR "protects"[All Fields])) OR (("economics"[MeSH Terms] OR "economics"[All Fields] OR "financial"[All Fields] OR "financially"[All Fields] OR "financials"[All Fields] OR "financier"[All Fields] OR "financiers"[All Fields]) AND ("risk"[MeSH Terms] OR "risk"[All Fields]) AND ("protect"[All Fields] OR "protected"[All Fields] OR "protecting"[All Fields] OR "protection"[All Fields] OR "protections"[All Fields] OR "protective agents"[Pharmacological Action] OR "protective agents"[MeSH Terms] OR ("protective"[All Fields] AND "agents"[All Fields]) OR "protective agents"[All Fields] OR "protectant"[All Fields] OR "protectants"[All Fields] OR "protective"[All Fields] OR "protectively"[All Fields] OR "protectiveness"[All Fields] OR "protectives"[All Fields] OR "protects"[All Fields])) OR (("catastrophe"[All Fields] OR "catastrophes"[All Fields] OR "catastrophic"[All Fields] OR "catastrophically"[All Fields]) AND ("health expenditures"[MeSH Terms] OR ("health"[All Fields] AND "expenditures"[All Fields]) OR "health expenditures"[All Fields] OR "expenditure"[All Fields] OR "expenditures"[All Fields])) OR (("catastrophe"[All Fields] OR "catastrophes"[All Fields] OR "catastrophic"[All Fields] OR "catastrophically"[All Fields]) AND ("health expenditures"[MeSH Terms] OR ("health"[All Fields] AND "expenditures"[All Fields]) OR "health expenditures"[All Fields] OR ("health"[All Fields] AND "expenditure"[All Fields]) OR "health expenditure"[All Fields])) OR (("adaptation, psychological"[MeSH Terms] OR ("adaptation"[All Fields] AND "psychological"[All Fields]) OR "psychological adaptation"[All Fields] OR "coping"[All Fields] OR "coped"[All Fields] OR "copes"[All Fields] OR "copings"[All Fields]) AND ("mechanism"[All Fields] OR "mechanisms"[All Fields])) OR ("financial stress"[MeSH Terms] OR ("financial"[All Fields] AND "stress"[All Fields]) OR "financial stress"[All Fields] OR ("financial"[All Fields] AND "burden"[All Fields]) OR "financial burden"[All Fields]) OR ("financial stress"[MeSH Terms] OR ("financial"[All Fields] AND "stress"[All Fields]) OR "financial stress"[All Fields] OR ("financial"[All Fields] AND "hardship"[All Fields]) OR "financial hardship"[All Fields]) OR ("out"[All Fields] AND ("pocket"[All Fields] OR "pocket s"[All Fields] OR "pocketing"[All Fields] OR "pockets"[All Fields])) OR "OOP"[All Fields] OR ("impoverish"[All Fields] OR "impoverished"[All Fields] OR "impoverishes"[All Fields] OR "impoverishing"[All Fields] OR "impoverishment"[All Fields]) OR ("health expenditures"[MeSH Terms] OR ("health"[All Fields] AND "expenditures"[All Fields]) OR "health expenditures"[All Fields] OR ("health"[All Fields] AND "expenditure"[All Fields]) OR "health expenditure"[All Fields]) OR (("health"[MeSH Terms] OR "health"[All Fields] OR "health s"[All Fields] OR "healthful"[All Fields] OR "healthfulness"[All Fields] OR "healths"[All Fields]) AND ("expense"[All Fields] OR "expenses"[All Fields] OR "expensive"[All Fields] OR "expensively"[All Fields])) OR (("medic"[All Fields] OR "medical"[All Fields] OR "medicalization"[MeSH Terms] OR "medicalization"[All Fields] OR "medicalizations"[All Fields] OR "medicalize"[All Fields] OR "medicalized"[All Fields] OR "medicalizes"[All Fields] OR "medicalizing"[All Fields] OR "medically"[All Fields] OR "medicals"[All Fields] OR "medicated"[All Fields] OR "medication s"[All Fields] OR "medics"[All Fields] OR "pharmaceutical preparations"[MeSH Terms] OR ("pharmaceutical"[All Fields] AND "preparations"[All Fields]) OR "pharmaceutical preparations"[All Fields] OR "medication"[All Fields] OR "medications"[All Fields]) AND ("expense"[All Fields] OR "expenses"[All Fields] OR "expensive"[All Fields] OR "expensively"[All Fields])) OR ("health expenditures"[MeSH Terms] OR ("health"[All Fields] AND "expenditures"[All Fields]) OR "health expenditures"[All Fields] OR ("health"[All Fields] AND "expenditure"[All Fields]) OR "health expenditure"[All Fields]) OR ("health care costs"[MeSH Terms] OR ("health"[All Fields] AND "care"[All Fields] AND "costs"[All Fields]) OR "health care costs"[All Fields] OR ("health"[All Fields] AND "cost"[All Fields]) OR "health cost"[All Fields]) OR (("medic"[All Fields] OR "medical"[All Fields] OR "medicalization"[MeSH Terms] OR "medicalization"[All Fields] OR "medicalizations"[All Fields] OR "medicalize"[All Fields] OR "medicalized"[All Fields] OR "medicalizes"[All Fields] OR "medicalizing"[All Fields] OR "medically"[All Fields] OR "medicals"[All Fields] OR "medicated"[All Fields] OR "medication s"[All Fields] OR "medics"[All Fields] OR "pharmaceutical preparations"[MeSH Terms] OR ("pharmaceutical"[All Fields] AND "preparations"[All Fields]) OR "pharmaceutical preparations"[All Fields] OR "medication"[All Fields] OR "medications"[All Fields]) AND ("economics"[MeSH Subheading] OR "economics"[All Fields] OR "cost"[All Fields] OR "costs and cost analysis"[MeSH Terms] OR ("costs"[All Fields] AND "cost"[All Fields] AND "analysis"[All Fields]) OR "costs and cost analysis"[All Fields])) OR ("copayment"[All Fields] OR "copayments"[All Fields])) AND ("gender identity"[MeSH Terms] OR ("gender"[All Fields] AND "identity"[All Fields]) OR "gender identity"[All Fields] OR "gendered"[All Fields] OR "gender s"[All Fields] OR "gendering"[All Fields] OR "genderized"[All Fields] OR "genders"[All Fields] OR "sex"[MeSH Terms] OR "sex"[All Fields] OR "gender"[All Fields] OR ("mother s"[All Fields] OR "mothered"[All Fields] OR "mothers"[MeSH Terms] OR "mothers"[All Fields] OR "mother"[All Fields] OR "mothering"[All Fields]) OR ("pregnancy"[MeSH Terms] OR "pregnancy"[All Fields] OR "pregnancies"[All Fields] OR "pregnancy s"[All Fields]) OR ("pregnant women"[MeSH Terms] OR ("pregnant"[All Fields] AND "women"[All Fields]) OR "pregnant women"[All Fields]) OR (("reproduction"[MeSH Terms] OR "reproduction"[All Fields] OR "reproductions"[All Fields] OR "reproductive"[All Fields] OR "reproductively"[All Fields] OR "reproductives"[All Fields] OR "reproductivity"[All Fields]) AND ("agrosyst geosci environ"[Journal] OR "age"[Journal] OR "age omaha"[Journal] OR "age dordr"[Journal] OR "adv genet eng"[Journal] OR "age"[All Fields])) OR (("child"[MeSH Terms] OR "child"[All Fields] OR "children"[All Fields] OR "child s"[All Fields] OR "children s"[All Fields] OR "childrens"[All Fields] OR "childs"[All Fields]) AND ("birth s"[All Fields] OR "birthed"[All Fields] OR "birthing"[All Fields] OR "parturition"[MeSH Terms] OR "parturition"[All Fields] OR "birth"[All Fields] OR "births"[All Fields])) OR ("maternal health"[MeSH Terms] OR ("maternal"[All Fields] AND "health"[All Fields]) OR "maternal health"[All Fields]) OR ("reproductive health"[MeSH Terms] OR ("reproductive"[All Fields] AND "health"[All Fields]) OR "reproductive health"[All Fields]) OR ("family planning services"[MeSH Terms] OR ("family"[All Fields] AND "planning"[All Fields] AND "services"[All Fields]) OR "family planning services"[All Fields] OR ("family"[All Fields] AND "planning"[All Fields]) OR "family planning"[All Fields]) OR ("contraception"[MeSH Terms] OR "contraception"[All Fields] OR ("birth"[All Fields] AND "control"[All Fields]) OR "birth control"[All Fields]) OR (("hospital s"[All Fields] OR "hospitalisation"[All Fields] OR "hospitalization"[MeSH Terms] OR "hospitalization"[All Fields] OR "hospitalised"[All Fields] OR "hospitalising"[All Fields] OR "hospitality"[All Fields] OR "hospitalisations"[All Fields] OR "hospitalizations"[All Fields] OR "hospitalize"[All Fields] OR "hospitalized"[All Fields] OR "hospitalizing"[All Fields] OR "hospitals"[MeSH Terms] OR "hospitals"[All Fields] OR "hospital"[All Fields]) AND ("deliveries"[All Fields] OR "delivery, obstetric"[MeSH Terms] OR ("delivery"[All Fields] AND "obstetric"[All Fields]) OR "obstetric delivery"[All Fields] OR "delivery"[All Fields])) OR ("contracept"[All Fields] OR "contracepted"[All Fields] OR "contracepting"[All Fields] OR "contraception"[MeSH Terms] OR "contraception"[All Fields] OR "contraceptions"[All Fields] OR "contraceptive agents"[Pharmacological Action] OR "contraceptive agents"[MeSH Terms] OR ("contraceptive"[All Fields] AND "agents"[All Fields]) OR "contraceptive agents"[All Fields] OR "contraceptives"[All Fields] OR "contraceptive devices"[MeSH Terms] OR ("contraceptive"[All Fields] AND "devices"[All Fields]) OR "contraceptive devices"[All Fields] OR "contraceptive"[All Fields] OR "contraceptive s"[All Fields] OR "contraceptively"[All Fields]) OR ("prenatal"[All Fields] OR "prenatally"[All Fields] OR "prenatals"[All Fields]) OR ("antenatal"[All Fields] OR "antenatally"[All Fields]) OR ("post"[All Fields] AND ("natal"[All Fields] OR "natally"[All Fields])) OR ("caesarean section"[All Fields] OR "cesarean section"[MeSH Terms] OR ("cesarean"[All Fields] AND "section"[All Fields]) OR "cesarean section"[All Fields]) OR (("assistances"[All Fields] OR "assistant s"[All Fields] OR "assistants"[All Fields] OR "assisted"[All Fields] OR "assisting"[All Fields] OR "assistive"[All Fields] OR "dental assistants"[MeSH Terms] OR ("dental"[All Fields] AND "assistants"[All Fields]) OR "dental assistants"[All Fields] OR "assistant"[All Fields] OR "helping behavior"[MeSH Terms] OR ("helping"[All Fields] AND "behavior"[All Fields]) OR "helping behavior"[All Fields] OR "assist"[All Fields] OR "assistance"[All Fields] OR "assists"[All Fields]) AND ("deliveries"[All Fields] OR "delivery, obstetric"[MeSH Terms] OR ("delivery"[All Fields] AND "obstetric"[All Fields]) OR "obstetric delivery"[All Fields] OR "delivery"[All Fields])) OR (("normal"[All Fields] OR "normalisation"[All Fields] OR "normalisations"[All Fields] OR "normalise"[All Fields] OR "normalised"[All Fields] OR "normalises"[All Fields] OR "normalising"[All Fields] OR "normalization"[All Fields] OR "normalizations"[All Fields] OR "normalize"[All Fields] OR "normalized"[All Fields] OR "normalizer"[All Fields] OR "normalizers"[All Fields] OR "normalizes"[All Fields] OR "normalizing"[All Fields] OR "normally"[All Fields] OR "normals"[All Fields]) AND ("deliveries"[All Fields] OR "delivery, obstetric"[MeSH Terms] OR ("delivery"[All Fields] AND "obstetric"[All Fields]) OR "obstetric delivery"[All Fields] OR "delivery"[All Fields])) OR ("sexual health"[MeSH Terms] OR ("sexual"[All Fields] AND "health"[All Fields]) OR "sexual health"[All Fields]) OR (("prevent"[All Fields] OR "preventability"[All Fields] OR "preventable"[All Fields] OR "preventative"[All Fields] OR "preventatively"[All Fields] OR "preventatives"[All Fields] OR "prevented"[All Fields] OR "preventing"[All Fields] OR "prevention and control"[MeSH Subheading] OR ("prevention"[All Fields] AND "control"[All Fields]) OR "prevention and control"[All Fields] OR "prevention"[All Fields] OR "prevention s"[All Fields] OR "preventions"[All Fields] OR "preventive"[All Fields] OR "preventively"[All Fields] OR "preventives"[All Fields] OR "prevents"[All Fields]) AND ("infectious disease transmission, vertical"[MeSH Terms] OR ("infectious"[All Fields] AND "disease"[All Fields] AND "transmission"[All Fields] AND "vertical"[All Fields]) OR "vertical infectious disease transmission"[All Fields] OR ("mother"[All Fields] AND "child"[All Fields] AND "transmission"[All Fields]) OR "mother to child transmission"[All Fields]) AND ("hiv"[MeSH Terms] OR "hiv"[All Fields])) OR ("perinatal"[All Fields] OR "perinatally"[All Fields] OR "perinatals"[All Fields]) OR ("safe"[All Fields] AND ("deliveries"[All Fields] OR "delivery, obstetric"[MeSH Terms] OR ("delivery"[All Fields] AND "obstetric"[All Fields]) OR "obs

**Embase search string**

#1 AND #2 AND #3 AND #4 AND #5

#5 'low income country':ti,ab,kw OR 'lower middle income country':ti,ab,kw OR 'low resource setting':ti,ab,kw OR 'developing country':ti,ab,kw OR 'africa south of the sahara':ti,ab,kw OR afghanistan:ti,ab,kw OR 'guinea bissau':ti,ab,kw OR somalia:ti,ab,kw OR 'burkina faso':ti,ab,kw OR 'north korean':ti,ab,kw OR 'south sudan':ti,ab,kw OR burundi:ti,ab,kw OR liberia:ti,ab,kw OR sudan:ti,ab,kw OR 'central african republic':ti,ab,kw OR madagascar:ti,ab,kw OR 'syrian arab republic':ti,ab,kw OR chad:ti,ab,kw OR malawi:ti,ab,kw OR togo:ti,ab,kw OR 'democratic republic congo':ti,ab,kw OR mali:ti,ab,kw OR uganda:ti,ab,kw OR eritrea:ti,ab,kw OR mozambique:ti,ab,kw OR yemen:ti,ab,kw OR ethiopia:ti,ab,kw OR niger:ti,ab,kw OR gambia:ti,ab,kw OR rwanda:ti,ab,kw OR guinea:ti,ab,kw OR 'sierra leone':ti,ab,kw OR angola:ti,ab,kw OR honduras:ti,ab,kw OR philippines:ti,ab,kw OR algeria:ti,ab,kw OR india:ti,ab,kw OR samoa:ti,ab,kw OR bangladesh:ti,ab,kw OR indonesia:ti,ab,kw OR ('sao tome':ti,ab,kw AND principe:ti,ab,kw) OR belize:ti,ab,kw OR iran:ti,ab,kw OR senegal:ti,ab,kw OR benin:ti,ab,kw OR kenya:ti,ab,kw OR 'solomon islands':ti,ab,kw OR bhutan:ti,ab,kw OR kiribati:ti,ab,kw OR 'sri lanka':ti,ab,kw OR bolivia:ti,ab,kw OR kyrgyzstan:ti,ab,kw OR tanzania:ti,ab,kw OR 'cape verde':ti,ab,kw OR laos:ti,ab,kw OR tajikistan:ti,ab,kw OR cambodia:ti,ab,kw OR lesotho:ti,ab,kw OR 'timor leste':ti,ab,kw OR cameroon:ti,ab,kw OR mauritania:ti,ab,kw OR tunisia:ti,ab,kw OR comoros:ti,ab,kw OR 'federated states of micronesia':ti,ab,kw OR ukraine:ti,ab,kw OR congo:ti,ab,kw OR mongolia:ti,ab,kw OR uzbekistan:ti,ab,kw OR 'cote d`ivoire':ti,ab,kw OR morocco:ti,ab,kw OR vanuatu:ti,ab,kw OR djibouti:ti,ab,kw OR myanmar:ti,ab,kw OR 'viet nam':ti,ab,kw OR egypt:ti,ab,kw OR nepal:ti,ab,kw OR 'gaza strip palestine':ti,ab,kw OR 'el salvador':ti,ab,kw OR nicaragua:ti,ab,kw OR zambia:ti,ab,kw OR eswatini:ti,ab,kw OR nigeria:ti,ab,kw OR zimbabwe:ti,ab,kw OR ghana:ti,ab,kw OR pakistan:ti,ab,kw OR haiti:ti,ab,kw OR 'papua new guinea':ti,ab,kw

#4 impact:ti,ab,kw OR 'effect size':ti,ab,kw OR epidemiology:ti,ab,kw OR affect:ti,ab,kw OR 'object relation':ti,ab,kw OR therapy:ti,ab,kw OR 'causal modeling':ti,ab,kw OR 'causal attribution':ti,ab,kw OR 'association'/exp OR 'association' OR 'association'/exp OR association

#3 insurance:ti,ab,kw OR 'government funded health insurance' OR 'health insurance'/syn OR 'health insurance' OR 'social insurance':ti,ab,kw OR 'national health insurance':ti,ab,kw OR 'community-based health insurance':ti,ab,kw OR 'public health insurance':ti,ab,kw OR 'regional health insurance':ti,ab,kw OR 'risk pooling':ti,ab,kw

#2 'health care utilization'/syn OR 'health care utilization' OR 'health care utilization'/exp OR 'health care utilization' OR 'health care access'/syn OR 'health care access' OR 'health care access'/exp OR 'health care access' OR 'health care':ti,ab,kw OR 'health service':ti,ab,kw OR 'financial protection' OR 'financial risk protection':ti,ab,kw OR 'catastrophic health expenditure':ti,ab,kw OR 'catastrophic expenditure' OR 'out of pocket expenditure':ti,ab,kw OR 'out of pocket payment':ti,ab,kw OR 'out of pocket cost':ti,ab,kw OR 'health care cost':ti,ab,kw OR 'medical cost':ti,ab,kw OR 'financial stress':ti,ab,kw OR 'financial burden':ti,ab,kw OR copayment:ti,ab,kw

#1 ‘'mother'/syn OR 'mother' OR 'mother'/exp OR 'mother' OR 'gender and sex'/syn OR 'gender and sex' OR 'pregnancy'/syn OR 'pregnancy' OR 'pregnancy'/exp OR pregnancy OR 'reproduction'/syn OR 'reproduction' OR 'reproduction'/exp OR reproduction OR childbirth:ti,ab,kw OR 'maternal care':ti,ab,kw OR 'maternal health service'/syn OR 'maternal health service' OR 'maternal health service'/exp OR 'maternal health service' OR 'health service':ti,ab,kw OR 'birth control'/syn OR 'birth control' OR 'birth control'/exp OR 'birth control' OR contraception:ti,ab,kw OR 'prenatal care':ti,ab,kw OR 'postnatal care':ti,ab,kw OR 'cesarean section':ti,ab,kw OR abortion:ti,ab,kw OR 'obstetric procedure':ti,ab,kw

**Web of Science search string**

**Platform:** Web of Science Core collection on Clarivate

**Search string**

1: (((((((((((((((TS =(Gender)) OR TS=(mother)) OR TS=(Pregnancy)) OR TS=(reproduction)) OR TS=(childbirth)) OR TS=(maternal care))) OR TS=(maternal health service)) OR TS=(health service)) OR TS=(birth control)) OR TS=(contraception)) OR TS=(prenatal care)) OR TS=(postnatal)) OR TS=(cesarean section)) OR TS=(abortion)) OR TS=(obstetric procedure) Date Run: Tue May 23 2023 10:53:00 GMT+0100 (British Summer Time) Results: 2252271

2: (((((((((((((((TS=(Health care utilisation)) OR TS=(health care access)) OR TS=(health care)) OR TS=(health service)) OR TS=(financial protection)) OR TS=(financial risk protection)) OR TS=(catastrophic health expenditure)) OR TS=(catastrophic expenditure)) OR TS=(out of pocket expenditure)) OR TS=(out of pocket payment)) OR TS=(out of pocket payment)) OR TS=(health care cost)) OR TS=(medical cost)) OR TS=(financial stress)) OR TS=(financial burden)) OR TS=(copayment) Date Run: Tue May 23 2023 10:53:55 GMT+0100 (British Summer Time) Results: 1143917

3: ((((((((TS =(Government funded health insurance)) OR TS=(Insurance)) OR TS=(Health insurance)) OR TS=(social insurance)) OR TS=(national health insurance)) OR TS=(community-based health insurance)) OR TS=(public health insurance)) OR TS=(regional health insurance)) OR TS=(risk pooling) Date Run: Tue May 23 2023 10:55:01 GMT+0100 (British Summer Time) Results: 224231

4: ((((((((TS=(impact)) OR TS=(effect size)) OR TS=(epidemiology)) OR TS=(affect)) OR TS=(object relation)) OR TS=(therapy)) OR TS=(causal modeling)) OR TS=(causal attribution)) OR TS=(association) Date Run: Tue May 23 2023 10:55:57 GMT+0100 (British Summer Time) Results: 12065253

5: (((((((((((((((((((((((((((((((((((((((((((((((((((((((((((((((((((((((((((((((((((((((TS=(low income country)) OR TS=(lower middle income country)) OR TS=(low resource setting)) OR TS=(developing country)) OR TS=(Africa south of the Sahara)) OR TS=(Afghanistan)) OR TS=(guinea bissau)) OR TS=(Somali)) OR TS=(Burkina Faso)) OR TS=(North Korea)) OR TS=(South Sudan)) OR TS=(Burundi)) OR TS=(Liberia)) OR TS=(Sudan)) OR TS=(Central African Republic)) OR TS=(Madagascar)) OR TS=(Syria)) OR TS=(Chad)) OR TS=(Malawi)) OR TS=(Togo)) OR TS=(democratic republic of Congo)) OR TS=(mali)) OR TS=(Uganda)) OR TS=(Eritrea)) OR TS=(Mozambique)) OR TS=(Yemen)) OR TS=(Ethiopia)) OR TS=(Niger)) OR TS=(Gambia)) OR TS=(Rwanda)) OR TS=(Guinea)) OR TS=(Sierra leone)) OR TS=(Angola)) OR TS=(Honduras)) OR TS=(Philippines)) OR TS=(Algeria)) OR TS=(India)) OR TS=(Samoa)) OR TS=(Bangladesh)) OR TS=(Indonesia)) OR TS=(Sao tome )) OR TS=(Belize)) OR TS=(Iran)) OR TS=(Senegal)) OR TS=(Benin)) OR TS=(Kenya)) OR TS=(Solomon Islands)) OR TS=(Bhutan)) OR TS=(Kiribati)) OR TS=(Sri Lanka)) OR TS=(Bolivia)) OR TS=(Kyrgyzstan)) OR TS=(Tanzania)) OR TS=(Cape Verde)) OR TS=(Laos)) OR TS=(Tajikistan)) OR TS=(Cambodia)) OR TS=(Lesotho)) OR TS=(Timor leste)) OR TS=(Cameroon)) OR TS=(Mauritania)) OR TS=(Tunisia)) OR TS=(Comoros)) OR TS=(Micronesia)) OR TS=(Ukraine)) OR TS=(Congo)) OR TS=(Mongolia)) OR TS=(Uzbekistan)) OR TS=(Cote d Ivoire)) OR TS=(Morocco)) OR TS=(Vanuatu)) OR TS=(Djibouti)) OR TS=(Myanmar)) OR TS=(Vietnam)) OR TS=(Egypt)) OR TS=(Nepal)) OR TS=(Gaza)) OR TS=(El salvador)) OR TS=(Nicaragua)) OR TS=(Zambia)) OR TS=(Eswatini)) OR TS=(Swaziland)) OR TS=(Nigeria)) OR TS=(Zimbabwe)) OR TS=(Ghana)) OR TS=(Pakistan)) OR TS=(haiti)) OR TS=(Papua New Guinea) Date Run: Tue May 23 2023 10:56:46 GMT+0100 (British Summer Time) Results: 1926007

6: (((((((((TS =(Government funded health insurance)) OR TS=(Insurance)) OR TS=(Health insurance)) OR TS=(social insurance)) OR TS=(national health insurance)) OR TS=(community-based health insurance)) OR TS=(public health insurance)) OR TS=(regional health insurance)) OR TS=(risk pooling) OR TS=(Mutuelles de Santé)) Date Run: Tue May 23 2023 10:59:40 GMT+0100 (British Summer Time) Results: 224232

7: ((((#1) AND #2) AND #3) AND #4) AND #5

**Scopus**

**Platform: Scopus**

**Search string**

( TITLE-ABS-KEY-AUTH ( mother OR gender OR pregnancy OR reproduction OR childbirth OR "maternal care" OR "maternal health service" OR "health service" OR "birth control" OR contraception OR "prenatal care" OR postnatal OR "cesarean section" OR abortion OR "obstetric procedure" ) ) AND ( TITLE-ABS-KEY-AUTH ( "Health care utilisation" OR "health care access" OR "health care" OR "health service" OR "financial protection" OR "financial risk protection" OR "catastrophic health expenditure" OR "catastrophic expenditure" OR "out of pocket expenditure" OR "out of pocket payment" OR "out of pocket payment" OR "health care cost" OR "medical cost" OR "financial stress" OR "financial burden" OR copayment ) ) AND ( TITLE-ABS-KEY-AUTH ( insurance OR "Health insurance" OR "social insurance" OR "national health insurance" OR "government funded health insurance" OR "community-based health insurance" OR "public health insurance" OR "regional health insurance" OR "risk pooling" ) ) AND ( TITLE-ABS-KEY-AUTH ( impact OR "effect size" OR epidemiology OR affect OR "object relation" OR therapy OR "causal modelling" OR "causal attribution" OR association ) ) AND ( TITLE-ABS-KEY-AUTH ( "low income country" ) OR TITLE-ABS-KEY ( "lower middle income country" ) OR TITLE-ABS-KEY ( developing AND country ) OR TITLE-ABS-KEY ( "low resource setting" OR "Africa south of the Sahara" OR afghanistan OR "guinea Bissau" OR somali OR "Burkina Faso" OR "North Korea" OR "south sudan" OR burundi OR liberia OR sudan OR "Central African Republic" OR madagascar OR syria OR chad OR malawi OR togo OR "democratic republic of Congo" OR mali OR uganda OR eritrea OR mozambique OR yemen OR ethiopia OR niger OR gambia OR rwanda OR guinea OR "Sierra leone" OR angola OR honduras OR philippines OR algeria OR india OR samoa OR bangladesh OR indonesia OR "Sao tome" OR belize OR iran OR senegal OR benin OR kenya OR "Solomon Islands" OR bhutan OR kiribati OR "sri lanka" OR bolivia OR kyrgyzstan OR tanzania OR "cape verde" OR laos OR tajikistan OR cambodia OR lesotho OR "Timor leste" OR cameroon OR mauritania OR tunisia OR comoros OR micronesia OR ukraine OR congo OR mongolia OR uzbekistan OR "Cote d Ivoire" OR morocco OR vanuatu OR djibouti OR myanmar OR vietnam OR egypt OR nepal OR gaza OR "El Salvador" OR nicaragua OR zambia OR eswatini OR swaziland OR nigeria OR zimbabwe OR ghana OR pakistan OR haiti OR "Papua New Guinea" ) )

**Appendix 2: Table showing the quality assessment of included articles using the ROBINS I tool by GRADE.**

| **Study** | **Bias due to confounding** | **Bias in selection of participants into the study** | **Bias in classification of interventions** | **Bias due to deviations from intended interventions** | **Bias due to missing data** | **Bias in measurement of outcomes** | **Bias in selection of the reported result** | **Overall bias** | **Initial level of certainty** | **Lowering or raising the level of certainty** | **Final rating of certainty level** |
| --- | --- | --- | --- | --- | --- | --- | --- | --- | --- | --- | --- |
| Samarakoon et al 2020 | Serious risk | Low risk | Low risk | Low risk | Low risk | Low risk | Moderate risk | Serious risk | High | Reduce 2 levels -1 | Very Low |
| Agbanyo et al 2021 | Moderate risk | Low risk | Low risk | Low risk | Low risk | Low risk | Moderate risk | Moderate risk | High | Reduce 1 level + 1 - 1 | Moderate |
| Ravit et al 2020 | Moderate risk | Low risk | Low risk | Low risk | Low risk | Low risk | Low risk | Moderate risk | High | Reduce 1 level + 1 | High |
| Chang et al 2018 | Moderate risk | Low risk | Low risk | Low risk | Low risk | Low risk | Low risk | Moderate risk | High | Reduce 1 level + 1 | High |
| Rashad et al 2019 | Moderate risk | Low risk | Low risk | Low risk | Low risk | Low risk | Low risk | Moderate risk | High | Reduce 1 level + 1 | High |
| Gouda et al 2016 | Moderate risk | Low risk | Low risk | Low risk | Low risk | Low risk | Low risk | Moderate risk | High | Reduce 1 level + 1 | High |
| Philibert et al 2017 | Moderate risk | Moderate risk | Low risk | Low risk | Low risk | Low risk | Moderate risk | Moderate risk | High | Reduce 1 level + 1 - 1 - 1 | Low |
| Anindya et al 2020 | Moderate risk | Low risk | Low risk | Low risk | Low risk | Low risk | Low risk | Moderate risk | High | Reduce 1 level + 1 | High |
| Kuwawenaruwa et al 2019 | Moderate risk | Low risk | Low risk | Low risk | Low risk | Serious risk | Low risk | Serious risk | High | Reduce 2 levels | Low |
| Aizawa 2019a | Moderate risk | Low risk | Low risk | Low risk | Low risk | Low risk | Low risk | Moderate risk | High | Reduce 1 level + 1 | High |
| Bonfrer et al 2016 | Moderate risk | Low risk | Low risk | Low risk | Low risk | Low risk | Low risk | Moderate risk | High | Reduce 1 level + 1 | High |
| El Omari et al 2021 | Moderate risk | Low risk | Low risk | Low risk | Low risk | Low risk | Low risk | Moderate risk | High | Reduce 1 level + 1 | High |
| Wang et al 2017a | Moderate risk | Low risk | Low risk | Low risk | Low risk | Low risk | Low risk | Moderate risk | High | Reduce 1 level + 1 | High |
| Kofinti et al 2022a | Moderate risk | Low risk | Low risk | Low risk | Low risk | Low risk | Moderate risk | Moderate risk | High | Reduce 1 level + 1 - 1 | Moderate |
| Bousmah et al 2022 | Moderate risk | Low risk | Low risk | Low risk | Low risk | Low risk | Low risk | Moderate risk | High | Reduce 1 level + 1 | High |
| Mussa et al 2023 | Moderate risk | Low risk | Low risk | Low risk | Low risk | Low risk | Low risk | Moderate risk | High | Reduce 1 level + 1 | High |
| Garg et al 2023 | Moderate risk | Low risk | Low risk | Low risk | Low risk | Low risk | Low risk | Moderate risk | High | Reduce 1 level + 1 | High |
